# Comparison of two algorithms of APTT-based lupus anticoagulant assay, two Dilute Russell viper venom time reagents, and silica clotting time

**DOI:** 10.1371/journal.pone.0352430

**Published:** 2026-06-26

**Authors:** Preechaya Wongkrajang, Titiwan Pientong, Ratchaneekorn Hanyongyuth, Panutsaya Tientadakul

**Affiliations:** 1 Department of Clinical Pathology, Faculty of Medicine Siriraj Hospital, Mahidol University, Bangkok, Thailand; 2 Blood Transfusion Center, Faculty of Medicine, Khon Kaen University, Khon Kaen, Thailand; Celal Bayar University: Manisa Celal Bayar Universitesi, TÜRKIYE

## Abstract

Lupus anticoagulants (LA) are heterogeneous antiphospholipid antibodies that interfere with phospholipid-dependent coagulation assays, resulting in considerable variability among detection methods. Although international guidelines recommend a stepwise approach incorporating screening, mixing, and confirmatory testing, integrated strategies omitting routine mixing studies are widely used. Different percentile-based cutoffs have been proposed for defining LA positivity.We retrospectively analyzed 135 citrated plasma samples requested for LA testing. Five LA detection approaches were evaluated: 4 integrated assays and 1 activated partial thromboplastin time (APTT)–based approach following the ISTH-recommended stepwise algorithm with a mixing study. The integrated assays comprised silica clotting time, dilute Russell viper venom time (dRVVT) using 2 different reagent systems, and an APTT-based assay. Precision studies and reference intervals were established, and LA positivity rates were compared using 97.5th and 99th percentile cutoffs. Inter-assay agreement and associations with anticardiolipin (aCL) and anti–β2-glycoprotein I (aβ2GPI) antiphospholipid antibodies were assessed. LA positivity rates varied across procedures (19.3%–35.6%) at the 97.5th percentile. Application of the 99th percentile decreased positivity for dRVVT-HemosIL and APTT-based assays. Positivity increased at the 97.5th percentile in patients tested according to ISTH indications, with minimal impact in noncompliant cases. Inter-assay agreement ranged from fair to substantial and was influenced by assay type and cutoff definition. APTT-based assays showed the strongest associations with aCL and aβ2GPI antibodies. LA detection is strongly influenced by assay selection and cutoff strategy. Positivity rate–based evaluation provides a practical framework for comparing LA assays in laboratory practice, particularly in the absence of a reference standard.

## Introduction

Lupus anticoagulants (LA) are heterogeneous antibodies directed against phospholipids and phospholipid-binding proteins, such as β_2_-glycoprotein I and prothrombin, on anionic phospholipid surfaces [1,2]. LA testing is also among the diagnostic laboratory criteria for systemic lupus erythematosus (SLE) [3] and antiphospholipid syndrome (APS) [4]. APS is an autoimmune disorder defined by arterial, venous, or microvascular thrombosis; obstetric complications; or other clinical features, such as cardiac valve involvement and thrombocytopenia, in the presence of persistent antiphospholipid antibodies [4]. The antiphospholipid antibodies include LA, anticardiolipin (aCL), and anti–β_2_-glycoprotein I (aβ_2_GPI). Unlike aCL and aβ_2_GPI, which are detected using calibrated and highly stable solid-phase assays, LA detection relies on phospholipid-dependent coagulation tests [4].

Although termed anticoagulants, LA paradoxically promote thrombosis in vivo, manifesting as the clinical features of APS. In vitro, however, LA bind to phospholipid-binding proteins, most notably aβ_2_GPI, on negatively charged phospholipid surfaces, thereby prolonging clot formation in phospholipid-dependent coagulation tests [5]. This phospholipid dependence forms the basis of laboratory detection of LA.

Multiple assays are available for LA detection, such as dilute Russel viper venom time (dRVVT), silica clotting time (SCT), activated partial thromboplastin time (APTT)–based assays, kaolin clotting time, and dilute prothrombin time. Because no single assay can detect all LA, current guidelines recommend using at least 2 assays based on different principles to maximize diagnostic sensitivity. Among these, dRVVT is considered the most robust assay, owing to its overall diagnostic performance, and is recommended as 1 of the 2 core tests for LA detection [6]. Nevertheless, dRVVT, APTT-based assays, and SCT remain susceptible to interference from certain anticoagulants, including vitamin K antagonists and direct oral anticoagulants [6].

LA testing is recommended to follow a 3-step procedure comprising screening, mixing, and confirmatory tests [7]. In the screening step, a prolonged clotting time is identified using a phospholipid-dependent assay. The mixing study then demonstrates the presence of an inhibitor, and the confirmatory test shows shortening of the prolonged clotting time after the addition of excess phospholipid [7]. The mixing test helps reduce false-negative results when a potent LA cannot be adequately neutralized by the confirmatory reagent’s phospholipid content, obscuring the difference between screening and confirmatory results. However, the mixing test can also produce false-negative results through dilutional effects, particularly in patients with weak LA [8].

Several professional organizations have issued guidelines for laboratory detection of LA. These include recommendations from the International Society on Thrombosis and Haemostasis (ISTH) Scientific and Standardization Committee (SSC), published in 2009 [9] and updated in 2020 [10]. The Clinical and Laboratory Standards Institute (CLSI) also published guidelines in 2014 [8].

Although these guidelines share common principles, they differ in several aspects of LA testing, including assay selection, diagnostic algorithms, reference interval establishment, and cutoff value selection. In particular, the ISTH SSC guidelines [10] recommend the 99th percentile as the diagnostic cutoff for LA positivity, whereas the CLSI guidelines [8] recommend a 97.5th percentile cutoff.

LA testing remains method-dependent, and no single assay is considered a definitive reference standard. In routine clinical practice, laboratories employ various assay principles and testing algorithms, often influenced by local resources, workflow considerations, and cost constraints. Although current ISTH guidance recommends a stepwise testing strategy incorporating screening, mixing, and confirmatory procedures, this approach is not uniformly adopted [11].

In particular, integrated LA testing systems, in which screening and confirmatory reagents are interpreted without mandatory mixing studies, are widely used in routine practice. This is largely driven by practical considerations, including reduced turnaround time, lower reagent consumption, and avoidance of the additional costs of mixing studies. As a result, substantial variation exists between guideline recommendations and real-world laboratory practice [11].

This study aimed to compare the performance of 5 LA testing methods employing different assay principles and cutoff definitions. Many laboratories routinely use integrated testing strategies despite guideline recommendations favoring stepwise procedures with mixing studies. Accordingly, we sought to evaluate the practical advantages and limitations of these approaches and to assess the impact of 97.5th versus 99th percentile cutoffs on LA interpretation.

## Materials and methods

### Samples

In the precision study, 2 commercial quality control materials were used: (1) LA negative and positive controls (HemosIL; Werfen) and (2) LA control plasma (Hyphen BioMed).

Reference intervals and cutoff values were established using plasma samples from at least 50 healthy adult donors aged 18 years or older. Sample collection, preparation, and testing procedures for healthy donors were identical to those used for patient samples. The testing was performed between October 31 and November 30, 2022. Written informed consent was obtained from healthy donors for the use of their samples in establishing reference intervals.

For the comparison of LA detection methods, this retrospective laboratory-based study included 135 patients with clinically suspected APS or SLE referred for LA testing. The testing was performed at the Department of Clinical Pathology, Faculty of Medicine Siriraj Hospital, Mahidol University, Bangkok, Thailand, between January 1 and July 31, 2023. All available citrated plasma samples were tested using each of the evaluated LA assays. Patients receiving anticoagulant therapy at the time of sampling, including vitamin K antagonists, unfractionated heparin, or low-molecular-weight heparin, direct oral anticoagulants (DOACs) were excluded. Clinical information, including age, sex, underlying diagnosis, and relevant APS- and SLE-related clinical manifestations, was reviewed retrospectively from electronic medical records. Patient classification was assessed according to the 2006 revised Sapporo criteria for APS [12], the 2023 ACR/EULAR classification criteria for APS [4], and the 2019 EULAR/ACR classification criteria for SLE [3]. Results of aCL IgG/IgM and aβ_2_GPI IgG/IgM from routine clinical testing were also reviewed. This retrospective study was conducted using fully anonymized laboratory data and did not involve any patient-identifiable information. The requirement for informed consent for patient samples was waived by the Institutional Review Board because residual specimens from routine clinical testing were used, and all data were anonymized prior to analysis, posing minimal risk to participants. Patient samples were collected from 1 January to 31 July 2023, and the dataset was accessed for research purposes on 19 October 2023.

The study protocol was approved by the Siriraj Institutional Review Board (SIRB), Faculty of Medicine Siriraj Hospital, Mahidol University (COA no. Si-715/2022).

### Blood Collection and Specimen Preparation

Peripheral venous blood samples were collected in 0.109 M trisodium citrate tubes (9:1 blood-to-anticoagulant ratio; BD Vacutainer Citrate Tubes, BD). Samples were double-centrifuged at 1900*g* for 15 minutes at room temperature to obtain platelet-poor plasma. Although a platelet count below 10 000/μL was observed after the first centrifugation in our routine coagulation tests, double centrifugation was performed as recommended by the ISTH SSC guidelines [10]. This additional step minimizes the risk of residual platelet interference with LA testing.

Plasma was then aliquoted into 2 portions. One aliquot was used immediately for routine LA testing at Siriraj Hospital. The second aliquot was stored at −70 °C until analysis with the comparison assays. Each sample was rapidly thawed at 37 °C in a water bath for 5 minutes immediately before testing.

### Reagents and Instruments

LA testing was performed using the following reagents. (1) SCT was assessed using HemosIL Silica Clotting Time reagent (Werfen). (2) Dilute Russell viper venom time was performed using HemosIL dRVVT Screen and dRVVT Confirm reagents (Werfen; hereafter, dRVVT-HemosIL). (3) Dilute Russell viper venom time was also performed using Hemoclot LA-S (Screen) and LA-C (Confirm) reagents (Hyphen BioMed; hereafter, dRVVT-Hemoclot). (4) APTT-based LA testing was performed using Cephen LS, an LA-sensitive reagent, for screening and Cephen reagent for confirmation (Hyphen BioMed; hereafter, APTT-based LA). All assays were performed according to the manufacturers’ instructions.

The following analytical instruments were used. The ACL Top 500 Coagulation Analyzer (Instrumentation Laboratory Company) was used for SCT and dRVVT-HemosIL assays. The Sysmex CS-2500 coagulation analyzer (Sysmex Corporation) was used for dRVVT-Hemoclot and APTT-based LA assays.

### Study design

#### Precision Study.

A precision study was performed according to the CLSI EP15-A3 guideline (User Verification of Precision and Estimation of Bias, Third Edition) [13]. Normal and abnormal quality control materials were analyzed in 5 replicates per day for 5 consecutive days for each reagent.

In cases where the CLSI EP 15-A3 verification criteria were not met, we applied an alternative acceptance approach based on allowable total error (%TEa), derived from published biological variation data and the Data Innovations allowable error database. This approach has been used in previous laboratory validation studies when manufacturer claims are not met, but analytical performance remains clinically acceptable.

### Reference Intervals and Cutoff Values

Reference intervals and cutoff values were established using plasma samples from at least 50 healthy adult donors. The reference interval for each assay was defined as the 2.5th–97.5th percentile under the CLSI 2014 guidelines [8] and as the 1st–99th percentile under the ISTH SSC 2020 guidelines [10]. Cutoff values were defined using both the 97.5th percentile (per CLSI 2014 [8]) and the 99th percentile (per ISTH SSC 2020 [10]).

LA results were interpreted as positive when the normalized screen-to-confirm ratio exceeded the assay-specific cutoff value. This ratio is obtained by dividing the LA screen ratio by the LA confirm ratio. The LA screen ratio is calculated as the patient’s screening clotting time (seconds) divided by the mean of the LA screening reference interval. The LA confirm ratio is calculated in the same manner using the patient’s confirmatory clotting time and the mean of the LA confirmatory reference interval.

The cutoff values for aCL and aβ_2_GPI antibodies were defined as follows. aCL IgG was considered negative at < 4.5 GPL U/mL, borderline at 4.5–6.5 GPL U/mL, and positive at > 6.5 GPL U/mL. aCL IgM was considered negative at < 12 MPL U/mL. For aβ_2_GPI, both IgG and IgM were classified as negative at < 20 RU/mL.

### Comparison of LA Detection

Five LA testing approaches were evaluated. These included SCT, dRVVT-HemosIL, dRVVT-Hemoclot, and APTT-based LA testing using Cephen reagents. All 4 methods were interpreted as integrated assays, in which screening and confirmatory results were assessed without routine mixing studies. In addition, an APTT-based LA testing strategy following the ISTH-recommended stepwise procedure was evaluated using the same Cephen reagents. This approach incorporated a 1:1 mixing study with commercial normal pooled plasma at the screening and confirmatory steps. Mixing studies were interpreted according to normalized screen/confirm ratio criteria described in the ISTH SSC 2020 guidance [10].

Because no single reference standard exists for LA detection, positivity rates were used as a pragmatic comparative measure rather than sensitivity or specificity.

The performance of each LA detection algorithm was assessed and compared across methods. In addition, the effect of cutoff selection was analyzed by comparing results obtained using the 97.5th and 99th percentile cutoffs.

The study population was divided into 2 groups. The first group comprised patients who underwent testing in accordance with the ISTH SSC 2020 guidelines for patient selection for LA testing [10]. The second group comprised patients tested without meeting these guideline-based indications.

### Statistical Analysis

Statistical analysis was performed using Microsoft Excel 2019 (Microsoft Corp., Redmond, WA, USA). Demographic and clinical data were analyzed using descriptive statistics.

Precision was expressed as the coefficient of variation. The LA positivity rate was calculated for each assay, and inter-method agreement was assessed using the Cohen kappa coefficient. Associations between LA positivity and aCL or aβ_2_GPI were evaluated using odds ratios with 95% CI. A *P* value ≤ 0.05 was considered statistically significant.

## Results

### Patient Characteristics

A total of 135 patients with suspected APS or SLE were included. Approximately 80% of cases met the ISTH 2020 guideline-based indications for LA testing. Females predominated in both groups, with a higher proportion in the compliant group (100/112, 89%) than in the noncompliant group (16/23, 70%). Patients in the noncompliant group were older than those in the compliant group (mean [SD] age, 49.1 [21.9] vs 38.6 [12.4] years).

Among compliant indications, the most frequent reasons for testing were SLE; ischemic stroke, transient ischemic attack, or other cerebral ischemia in patients younger than 50 years; and infertility. In the noncompliant group, the most common reasons were testing in the absence of confirmed venous thromboembolism; and ischemic stroke or transient ischemic attack in patients aged 50 years or older (Table 1).

**Table 1:** Clinical indications for LA testing classified by guideline compliance (*N* = 135).

Clinical indication	*n*	%
**Guideline-compliant indications (*n* = 112)**		
Antiphospholipid syndrome	2	2
Arterial thrombosis in patients younger than 50 years	1	1
HELLP syndrome	1	1
Immune thrombocytopenia	1	1
Infertility	23	21
Ischemic stroke, TIA, or other cerebral ischemia in patients younger than 50 years	25	22
Livedo vasculopathy	3	3
Mixed connective tissue disease	1	1
Recurrent pregnancy loss	9	8
Rheumatoid arthritis	1	1
Systemic lupus erythematosus	34	30
Unprovoked VTE in patients younger than 50 years	5	4
Unspecified autoimmune disease	1	1
VTE at unusual sites	5	4
**Non-guideline-compliant indications (*n* = 23)**		
Ischemic stroke or TIA in patients aged 50 years or older	7	30
Local atherosclerosis	1	4
No definite confirmation for VTE	10	43
Other cerebral disease	1	4
Portal vein thrombosis in cirrhosis	1	4
Superficial vein thrombosis	1	4
VTE in patients aged 50 years or older with malignancy	2	9

Percentages are calculated within each compliance group (guideline-compliant, *n* = 112; non-guideline-compliant, *n* = 23). Compliance classification was based on established clinical criteria for appropriate LA testing per the ISTH SSC 2020 guidelines.

**Abbreviations:** HELLP, hemolysis, elevated liver enzymes, and low platelets; ISTH SSC, International Society on Thrombosis and Haemostasis Scientific and Standardization Committee; LA, lupus anticoagulant; TIA, transient ischemic attack; VTE, venous thromboembolism.

### Precision Study

Overall, precision performance was acceptable across assays, with low observed imprecision in most conditions. For SCT and dRVVT-HemosIL screen and confirm assays, repeatability and within-laboratory coefficient of variation values were consistently low at both normal and abnormal levels. However, several precision parameters for dRVVT-Hemoclot and APTT-based LA did not meet the manufacturer-recommended acceptance criteria.

Precision acceptability was therefore assessed using the percentage of total allowable error (%TEa) derived from the Allowable Total Error Table (Data Innovations, LLC) for APTT [14] and from the study by Kristoffersen et al [15]. On the basis of these %TEa limits, both repeatability and within-laboratory precision were considered acceptable (Table 2).

**Table 2:** Precision verification results for LA detection assays using the CLSI EP15-A3 protocol.

Assay	Control level	Repeatability						Within-laboratory reproducibility					
		s	%CVma	%UVL	CLSI result	%TEa/4	%TEa result	%CV	%CVma	%UVL	CLSI result	%TEa/3	%TEa result
SCT screen	Normal	1.7	6	—	Pass	—	—	2.3	6	—	Pass	—	—
	Abnormal	1.7	6	—	Pass	—	—	6	6	—	Pass	—	—
SCT confirm	Normal	1.4	6	—	Pass	—	—	2.2	6	—	Pass	—	—
	Abnormal	1.5	6	—	Pass	—	—	1.7	6	—	Pass	—	—
dRVVT-HemosIL screen	Normal	1.3	5	—	Pass	—	Pass	1.6	5	—	Pass	—	—
	Abnormal	2.8	5	—	Pass	—	Pass	4.5	5	—	Pass	—	—
dRVVT-HemosIL confirm	Normal	2.3	5	—	Pass	—	Pass	2.7	5	—	Pass	—	—
	Abnormal	3.1	5	—	Pass	—	Pass	3.7	5		Pass	—	—
dRVVT-Hemoclot screen	Normal	1.2	0.6	0.8	Fail	3.3	Pass	1.4	2.1	3.5	Pass	4.3	Pass
	Weak positive	1.2	0.6	0.8	Fail	3.3	Pass	1.8	4.0	6.6	Pass	4.3	Pass
	High positive	1.5	1.2	1.7	Pass	3.3	Pass	3.1	1.4	1.9	Fail	4.3	Pass
dRVVT-Hemoclot confirm	Normal	1.3	0.6	0.8	Fail	3.3	Pass	1.7	2.1	3.5	Pass	4.3	Pass
	Weak positive	0.9	0.5	0.7	Fail	3.3	Pass	1.4	0.7	1.0	Fail	4.3	Pass
	High positive	1.1	0.7	0.9	Fail	3.3	Pass	1.8	0.9	1.3	Fail	4.3	Pass
APTT-based LA screen	Normal	0.5	0.3	0.4	Fail	3.75	Pass	0.6	0.9	1.4	Pass	5	Pass
	Abnormal	0.4	0.3	0.4	Pass	3.75	Pass	0.7	0.8	1.3	Pass	5	Pass
APTT-based LA confirm	Normal	0.2	0.3	0.4	Pass	3.75	Pass	0.6	1.4	2.2	Pass	5	Pass
	Abnormal	0.09	0.3	0.4	Pass	3.75	Pass	0.1	0.9	1.4	pass	5	Pass

Precision was evaluated per CLSI EP15-A3 using 5 replicates per day for 5 consecutive days. The CLSI verification criterion requires %CV ≤ %CV_ma_ (manufacturer’s allowable %CV). When this criterion was not met, the upper verification limit (%UVL) was calculated and compared with %CV_ma_. If the %UVL criterion was also not met, a secondary criterion based on biologically derived %TEa was applied: %CV ≤ %TEa/4 for repeatability and %CV ≤ %TEa/3 for within-laboratory reproducibility. Dashes (—) indicate that the CLSI criterion was met and no further evaluation was required, or that no manufacturer’s claim was available.

**Abbreviations:** APTT, activated partial thromboplastin time; CLSI, Clinical and Laboratory Standards Institute; %CV, percentage coefficient of variation; %CV_ma_, manufacturer’s allowable coefficient of variation; dRVVT, dilute Russell viper venom time; LA, lupus anticoagulant; SCT, silica clotting time; %TEa, percentage of total allowable error; %UVL, percentage upper verification limit.

### Reference Intervals and Cutoff Values

Reference intervals and cutoff values were established from at least 50 healthy donors using both the 99th percentile (ISTH 2020 approach) and the 97.5th percentile (CLSI approach).

Although ISTH recommends ≥120 healthy individuals for establishing reference intervals, smaller cohorts are commonly used in laboratory verification studies and were considered acceptable for this exploratory comparison. Cutoff values differed between the 2 approaches and varied by assay and study period (January–March 2023 vs March–July 2023). As expected, the 99th percentile approach yielded more stringent upper limits than the 97.5th percentile approach. These assay-specific cutoffs were subsequently applied to determine LA positivity using normalized ratios (Table 3).

**Table 3:** Reference intervals and cutoff values for LA detection assays at the 99th and 97.5th percentiles.

Test	ISTH 2020 guideline (99th percentile)			CLSI guideline (97.5th percentile)		
	Reference interval	Cutoff, (s)	Cutoff (ratio)	Reference interval	Cutoff, (s)	Cutoff (ratio)
**January–March 2023, *N* = 50**						
SCT screen, s	33.67–61.88	61.88	—	33.67–59.93	59.93	—
SCT screen ratio	0.72–1.32	—	1.32	0.72–1.27	—	1.27
SCT confirm, s	32.83–57.13	57.13	—	32.83–56.16	56.16	—
SCT confirm ratio	0.77–1.34	—	1.34	0.77–1.32	—	1.32
SCT screen/confirm ratio	0.74–1.15	—	1.15	0.74–1.15	—	1.15
dRVVT-HemosIL screen, s	36.95–51.91	51.91	—	36.95–51.50	—	51.50
dRVVT-HemosIL screen ratio	0.84–1.18	—	1.18	0.84–1.17	—	1.17
dRVVT-HemosIL confirm, s	29.45–39.06	39.06	—	29.45–38.57	—	38.57
dRVVT-HemosIL confirm ratio	0.92–1.22	—	1.22	0.92–1.21	—	1.21
dRVVT-HemosIL screen/confirm ratio	0.87–1.16	—	1.16	0.87–1.15	—	1.15
**March–July 2023, *N* = 66**						
SCT screen, s	32.50–54.36	54.36	—	32.50–53.06	53.06	—
SCT screen ratio	0.75–1.25	—	1.25	0.75–1.22	—	1.22
SCT confirm, s	34.64–56.22	56.22	—	34.64–52.88	52.88	—
SCT confirm ratio	0.79–1.29	—	1.29	0.79–1.21	—	1.21
SCT screen/confirm ratio	0.83–1.22	—	1.22	0.83–1.20	—	1.20
dRVVT-HemosIL screen, s	25.61–43.65	43.65	—	25.61–42.13	42.13	—
dRVVT-HemosIL screen ratio	0.75–1.28	—	1.28	0.75–1.23	—	1.23
dRVVT-HemosIL confirm, s	25.93–34.00	34.00	—	25.93–33.73	33.73	—
dRVVT-HemosIL confirm ratio	0.88–1.16	—	1.16	0.88–1.15	—	1.15
dRVVT-HemosIL screen/confirm ratio	0.81–1.18	—	1.18	0.81–1.15	—	1.15
**January–July 2023, *N* = 120**						
dRVVT-Hemoclot screen, s	28.44–49.85	49.85	—	28.44–48.22	48.22	—
dRVVT-Hemoclot screen ratio	0.76–1.33	—	1.33	0.76–1.29	—	1.29
dRVVT-Hemoclot confirm, s	28.48–37.56	37.56	—	28.48–36.91	36.91	—
dRVVT-Hemoclot confirm ratio	0.88–1.16	—	1.16	0.88–1.14	—	1.14
dRVVT-Hemoclot screen/confirm ratio	0.82–1.23	—	1.23	0.82–1.22	—	1.22
APTT-based LA screen, s	31.92–49.17	49.17	—	31.92–48.50	48.50	—
APTT-based LA screen ratio	0.82–1.27	—	1.27	0.82–1.25	—	1.25
APTT-based LA confirm, s	28.98–42.98	42.98	—	28.98–41.91	41.91	—
APTT-based LA confirm ratio	0.85–1.29	—	1.29	0.85–1.22	—	1.22
APTT-based LA screen/confirm ratio	0.88–1.23	—	1.23	0.88–1.16	—	1.16
APTT-based LA mix screen, s	34.14–42.67	42.67	—	34.14–41.91	41.91	—
APTT-based LA mix screen ratio	0.91–1.13	—	1.13	0.91–1.10	—	1.10
APTT-based LA mix confirm, s	29.14–36.99	36.99	—	29.14–35.22	35.22	—
APTT-based LA mix confirm ratio	0.91–1.15	—	1.15	0.91–1.10	—	1.10
APTT-based LA mix screen/confirm ratio	0.93–1.09	—	1.09	0.93–1.08	—	1.08

Reference intervals were established using healthy donor samples collected during the indicated periods. Cutoff values represent the upper limit of the reference interval at the specified percentile. Dashes (—) indicate that the cutoff is not applicable for that parameter type (i.e., seconds-based cutoffs are reported for clotting time measurements and ratio-based cutoffs are reported for ratio calculations). “Mix” parameters refer to 1:1 mixing with normal pooled plasma per the ISTH SSC stepwise algorithm.

**Abbreviations:** APTT, activated partial thromboplastin time; CLSI, Clinical and Laboratory Standards Institute; dRVVT, dilute Russell viper venom time; ISTH, International Society on Thrombosis and Haemostasis; LA, lupus anticoagulant; s, seconds; SCT, silica clotting time.

### Comparison of LA Detection

Using the 97.5th percentile cutoff, total positivity varied across methods, ranging from 19.3% to 35.6%. The highest overall positivity was observed with dRVVT-HemosIL (48/135, 35.6%), whereas the lowest was observed with APTT-based LA (ISTH); 26/135, 19.3%). Applying the 99th percentile cutoff reduced total positivity for some assays, most notably for dRVVT-HemosIL (from 35.6% to 31.9%) and APTT-based LA (from 25.2% to 20.8%). APTT-based LA (ISTH) showed a smaller decrease (from 19.3% to 17.7%). SCT and dRVVT-Hemoclot positivity rates remained unchanged (Table 4).

**Table 4:** LA positivity rates by assay method and percentile cutoff according to guideline compliance (*N* = 135).

Assay method	Percentile cutoff	Guideline-compliant positive *n* (%)	Non-guideline-compliant positive *n* (%)	Total positive *n* (%)
SCT	97.5th	30 (22.2)	5 (3.7)	35 (25.9)
	99th	30 (22.2)	5 (3.7)	35 (25.9)
dRVVT-HemosIL	97.5th	40 (29.6)	8 (5.9)	48 (35.6)
	99th	35 (25.9)	8 (5.9)	43 (31.9)
dRVVT-Hemoclot	97.5th	34 (25.2)	2 (1.5)	36 (26.7)
	99th	34 (25.2)	2 (1.5)	36 (26.7)
APTT-based LA	97.5th	31 (23.0)	3 (2.2)	34 (25.2)
	99th	26 (19.3)	2 (1.5)	28 (20.8)
APTT-based LA (ISTH)	97.5th	24 (17.8)	2 (1.5)	26 (19.3)
	99th	23 (17.0)	1 (0.7)	24 (17.7)

Percentages are calculated from the total study population (*N* = 135; guideline-compliant, *n* = 112; non-guideline-compliant, *n* = 23). APTT-based LA refers to the integrated screen/confirm interpretation without a mixing step. APTT-based LA (ISTH) refers to the ISTH SSC 2020 stepwise algorithm incorporating a 1:1 mixing test with normal pooled plasma.

**Abbreviations:** APTT, activated partial thromboplastin time; dRVVT, dilute Russell viper venom time; ISTH, International Society on Thrombosis and Haemostasis; LA, lupus anticoagulant; SCT, silica clotting time.

**Fig 1:**
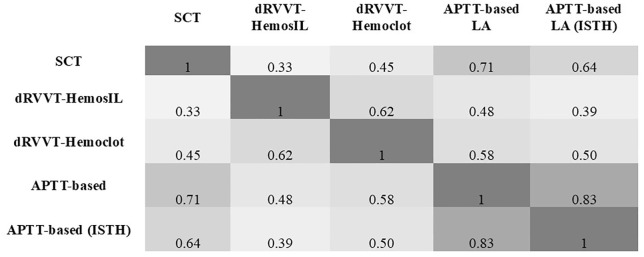
Heatmap of pairwise Cohen kappa agreement between 5 LA detection assays at the 97.5th percentile cutoffs. Each cell displays the Cohen kappa coefficient (κ) for pairwise agreement between the row and column assays in classifying specimens as LA positive or LA negative. Diagonal cells (—) represent self-agreement and are excluded. The matrix is symmetric; values above and below the diagonal are identical. Kappa values are interpreted as follows: less than 0.20, poor agreement; 0.20–0.39, fair agreement; 0.40–0.59, moderate agreement; 0.60–0.79, substantial agreement; and 0.80 or greater, excellent agreement. Cell shading reflects the strength of agreement. Values representing substantial or excellent agreement (κ ≥ 0.75) are shown in bold. APTT-based LA refers to the integrated screen/confirm interpretation without a mixing step; APTT-based LA (ISTH) refers to the ISTH SSC 2020 stepwise algorithm incorporating a 1:1 mixing test with normal pooled plasma. **Abbreviations:** APTT, activated partial thromboplastin time; dRVVT, dilute Russell viper venom time; ISTH, International Society on Thrombosis and Haemostasis; LA, lupus anticoagulant; SCT, silica clotting time; SSC, Scientific and Standardization Committee.

Pairwise agreement between assays ranged from fair to excellent and depended on the cutoff applied. At the 97.5th percentile cutoff, kappa values ranged from approximately 0.33 to 0.83, whereas at the 99th percentile cutoff, values ranged from approximately 0.39 to 0.90. The highest agreement was observed between the 2 APTT-based approaches, whereas lower agreement was seen between SCT and dRVVT-HemosIL. In addition, the sensitivity of method concordance to cutoff strategy varied by assay; dRVVT-Hemoclot showed minimal change in agreement across cutoffs (Fig 1).

At the 97.5th percentile cutoff, LA positivity was associated with aCL antibodies when detected by SCT, the APTT-based LA assay, and the APTT-based (ISTH) procedure, with odds ratios ranging from 14.52 to 23.78. The dRVVT-Hemoclot assay also showed an association with aCL positivity, though with a lower odds ratio. In contrast, the association for dRVVT-HemosIL did not reach statistical significance. At the 99th percentile cutoff, similar patterns were observed, with SCT and APTT-based assays yielding the largest odds ratios and dRVVT-HemosIL remaining nonsignificant.

For aβ₂GPI antibodies at the 97.5th percentile cutoff, the APTT-based methods and the APTT-based (ISTH) procedure yielded the largest odds ratios. SCT and dRVVT-based assays also showed statistically significant associations, with odds ratios ranging from 6.40 to 7.26. At the 99th percentile cutoff, odds ratios decreased for the APTT-based methods but increased for dRVVT-HemosIL (Table 5).

**Table 5:** Association between LA positivity and solid-phase antiphospholipid antibodies by assay method and percentile cutoff.

LA assay method	LA + /AB+	LA − /AB+	LA + /AB−	LA − /AB−	OR (95% CI)
**97.5th percentile cutoff**					
***Anticardiolipin antibodies***					
SCT	8	2	27	98	14.52 (2.9–72.4)
dRVVT-HemosIL	6	4	42	83	2.96 (0.8–11.1)
dRVVT-Hemoclot	7	3	29	96	7.72 (1.9–31.8)
APTT-based LA	8	2	26	99	15.23 (3–76.1)
APTT-based LA (ISTH)	8	2	18	107	23.78 (4.7-121.1)
***Anti-β***_***2***_ ***-glycoprotein I antibodies***					
SCT	6	3	29	97	6.69 (1.6–28.4)
dRVVT-HemosIL	7	2	41	85	7.26 (1.4–36.5)
dRVVT-Hemoclot	6	3	30	96	6.40 (1.5–27.2)
APTT-based LA	7	2	27	99	12.83 (2.5–65.4)
APTT-based LA (ISTH)	7	2	19	107	19.71 (3.8 - 102.2)
**99th percentile cutoff**					
***Anticardiolipin antibodies***					
SCT	8	2	27	98	14.52 (2.9–72.4)
dRVVT-HemosIL	6	4	37	88	3.57 (1.0–13.4)
dRVVT-Hemoclot	7	3	29	96	7.72 (1.9–31.8)
APTT-based LA	9	1	26	99	34.26 (4.2-282.9)
APTT-based LA (ISTH)	8	2	16	109	27.25 (5.3-139.9)
***Anti-β***_***2***_ ***-glycoprotein I antibodies***					
SCT	6	3	29	97	6.69 (1.6–28.4)
dRVVT-HemosIL	7	2	36	90	8.75 (1.70–44.1)
dRVVT-Hemoclot	6	3	30	96	6.40 (1.5–27.2)
APTT-based LA	7	2	28	98	12.25(2.41-62.3)
APTT-based LA (ISTH)	6	3	18	108	12(2.8-52.3)

Data are presented as 2 × 2 contingency counts. LA + /Ab+ indicates patients positive for both the specified LA assay and the solid-phase antibody test; LA − /Ab+ indicates patients negative for the LA assay but positive for the antibody test; and so on. Odds ratios and 95% confidence intervals were calculated to quantify the strength of association between LA positivity and each solid-phase antibody. APTT-based LA refers to the integrated screen/confirm interpretation without a mixing step; APTT-based LA (ISTH) refers to the ISTH SSC 2020 stepwise algorithm incorporating a 1:1 mixing test with normal pooled plasma.

**Abbreviations:** Ab, antibody; APTT, activated partial thromboplastin time; CI, confidence interval; dRVVT, dilute Russell viper venom time; ISTH, International Society on Thrombosis and Haemostasis; LA, lupus anticoagulant; OR, odds ratio; SCT, silica clotting time

LA positivity was associated with both aCL and aβ₂GPI across the LA assays evaluated at both the 97.5th and 99th percentile cutoffs. Odds ratios varied among assays but were generally greater than 1; however, the 95% confidence intervals were wide and largely overlapping, limiting confident differentiation between assays (Table 5).

## Discussion

This study evaluated the performance of 5 LA detection methods with respect to precision, reference intervals, positivity rates, inter-assay agreement, and clinical-serological associations across different assay designs and cutoff definitions.

Precision analysis showed that all compared methods met acceptability criteria when assessed using biologically derived %TEa (Table 2). In this study, some assays did not meet manufacturer or CLSI EP15-A3 verification criteria. In such cases, performance was further evaluated using biologically derived %TEa limits. In cases where CLSI criteria were not met, %TEa-based evaluation was used as a clinically relevant alternative, although this approach should be interpreted with caution due to limited standardization.

In this study, the 97.5th and 99th percentile cutoffs were compared using positivity rates rather than sensitivity, given the absence of a true reference standard for LA detection. The choice of cutoff value directly impacted on LA positivity rates. Using the 97.5th percentile cutoff increased detection rates for dRVVT-Hemosil, APTT-based LA, and APTT-based LA (ISTH); however, positivity rates remained unchanged for SCT and dRVVT-Hemoclot (Table 4). Tripodi et al. found that a lower percentile cutoff yielded a higher detection rate; however, that study compared dRVVT and APTT-based methods at the 99th and 95th percentiles (16). A large algorithm comparison study examined the 97.5^th^ vs. 99^th^ percentile cutoffs but did not report the sensitivity and specificity of each test. Notably, all 188 patients with persistent LA positivity by the CLSI 2014 guideline (using 97.5th percentile cutoffs), which served as the reference method in that study, were also positive under the ISTH SCC 2020 guideline algorithm (using 99th percentile cutoffs) (1). Regarding algorithm performance, the integrated APTT-based LA approach yielded a higher positivity rate than the ISTH stepwise algorithm, consistent with the findings of Lickteig et al [16].

However, the increased positivity at the 97.5th percentile was observed predominantly in the guideline-compliant group, with only minimal changes in the noncompliant group (Table 4). This finding suggests that the lower cutoff did not substantially increase nonspecific positivity. Taken together, these results indicate that evaluating cutoff effects using positivity rates provides a practical perspective on LA testing. Although the 99th percentile offers a more conservative threshold, the 97.5th percentile appears to align more closely with routine clinical practice by facilitating detection of additional LA-positive cases among appropriately selected patients.

An important finding is the difference between the integrated APTT-based approach and the ISTH stepwise algorithm when using the same reagent pair. The integrated strategy yielded higher LA positivity than the ISTH algorithm. This difference is consistent with the expected effect of incorporating a mixing study, which can reduce apparent inhibitor activity through dilution, particularly in weak LA, thereby shifting results below the decision threshold. Conversely, the stepwise approach may improve specificity by requiring inhibitor behavior in the mixing test, at the expense of sensitivity for low-titer inhibitors. Our data support this trade-off: when the ISTH algorithm was applied, positivity decreased modestly relative to the integrated interpretation, whereas agreement between the 2 APTT-based approaches remained the highest among all method pairs.

**Fig 2:**
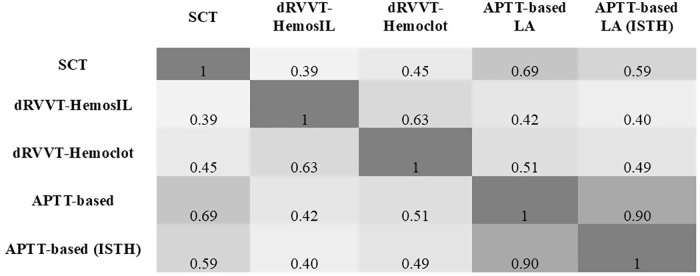
Heatmap of pairwise Cohen kappa agreement between 5 LA detection assays at the 99th percentile cutoffs. Each cell displays the Cohen kappa coefficient (κ) for pairwise agreement between the row and column assays in classifying specimens as LA positive or LA negative. Diagonal cells (—) represent self-agreement and are excluded. The matrix is symmetric; values above and below the diagonal are identical. Kappa values are interpreted as follows: less than 0.20, poor agreement; 0.20–0.39, fair agreement; 0.40–0.59, moderate agreement; 0.60–0.79, substantial agreement; and 0.80 or greater, excellent agreement. Cell shading reflects the strength of agreement. Values representing substantial or excellent agreement (κ ≥ 0.75) are shown in bold. APTT-based LA refers to the integrated screen/confirm interpretation without a mixing step; APTT-based LA (ISTH) refers to the ISTH SSC 2020 stepwise algorithm incorporating a 1:1 mixing test with normal pooled plasma. **Abbreviations:** APTT, activated partial thromboplastin time; dRVVT, dilute Russell viper venom time; ISTH, International Society on Thrombosis and Haemostasis; LA, lupus anticoagulant; SCT, silica clotting time; SSC, Scientific and Standardization Committee.

The heatmap analysis showed moderate agreement among the 5 LA assays at both the 97.5th and 99th percentile cutoffs. The highest agreement was observed between APTT-based methods, whereas agreement between SCT and dRVVT-based assays was lower, reflecting methodological differences and antibody heterogeneity. Increasing the cutoff to the 99th percentile slightly improved agreement in some assay pairs but did not change the overall pattern (Figs 1 and 2).

The association between LA positivity and solid-phase antiphospholipid antibodies varied according to the assay used and the cutoff applied. SCT also demonstrated a strong association with aCL antibodies at both percentile cutoffs, whereas its association with aβ_2_GPI was less pronounced. In contrast, dRVVT-HemosIL showed weaker associations with antibodies, especially at the 97.5th percentile, whereas dRVVT-Hemoclot yielded intermediate results. These findings suggest that reagent composition and interpretation strategy influence correlations with antibodies.

LA positivity was generally associated with aCL and aβ2GPI across most assays; however, some associations, particularly for dRVVT-HemosIL with aCL, did not reach statistical significance.

Although the estimated odds ratios differed among assays, the wide and overlapping 95% confidence intervals indicate substantial uncertainty, and no definitive differences between assays can be inferred (Table 5). Evidence from Nonobe et al demonstrated that LA assays and solid-phase antibodies may capture partly distinct risk indicators [17]. Discordance between LA positivity and aCL or aβ_2_GPI is therefore expected in practice and should be interpreted in the context of testing strategy and patient selection. These findings indicate that LA assays and solid-phase antiphospholipid antibody tests capture overlapping but not identical antibody populations. Differences in phospholipid composition, cofactor dependence, and assay design likely contribute to variable correlations between LA positivity and aCL or aβ_2_GPI antibodies across methods [17].

Overall, our findings highlight that algorithm choice (integrated vs stepwise with mixing) is not a purely technical preference but a determinant of positivity rates and method concordance. Laboratories should explicitly define and communicate their interpretive algorithm, particularly when comparing results across platforms or monitoring longitudinal LA status.

Study limitations included the absence of repeat LA testing. Current guidelines recommend confirmation of LA positivity on at least 2 occasions separated by 12 weeks or more; therefore, transient LA positivity cannot be excluded. In addition, some samples were obtained during acute thrombotic episodes, a setting known to interfere with coagulation assays and potentially produce both false-positive and false-negative LA results [10]. The relatively limited sample size may also have reduced statistical power for certain subgroup analyses and the precision of agreement estimates. These factors may have influenced LA positivity rates and assay concordance. Further studies incorporating larger cohorts and repeat testing are needed to confirm these findings.

## Conclusion

This study provides comparative data on the performance of 5 LA detection methods and demonstrates that assay selection and cutoff strategy substantially influence LA positivity rates. These findings highlight important analytical differences among methods and underscore the importance of method-specific interpretation when applying guideline recommendations in routine laboratory practice.

## Supporting information

S1 File Raw data. (PDF)

## Abbreviations

HELLP: hemolysis, elevated liver enzymes, and low platelets
ISTH SSC: International Society on Thrombosis and Haemostasis Scientific and Standardization Committee
LA: lupus anticoagulant
TIA: transient ischemic attack
VTE: venous thromboembolism;
APTT: activated partial thromboplastin time
CLSI: Clinical and Laboratory Standards Institute
%CV: percentage coefficient of variation
%CV_ma_: manufacturer’s allowable coefficient of variation
dRVVT: dilute Russell viper venom time
LA: lupus anticoagulant
SCT: silica clotting time
%TEa: percentage of total allowable error
%UVL: percentage upper verification limit: Ab, antibody
