# Supplementary material for: Comparison of two algorithms of APTT-based lupus anticoagulant assay, two Dilute Russell viper venom time reagents, and silica clotting time
Source: PLoS One. 2026 Jun 26;21(6):e0352430. doi: 10.1371/journal.pone.0352430 (PMC13308853; doi:10.1371/journal.pone.0352430)
Supplement: S1 File — (PDF) [file pone.0352430.s001.pdf]

No.	anti-cardiolipin	anti-beta2 glyclo protein I	Sex	Age	indications for testing
1	neg	neg	M	36	other brain disease
2	neg	neg	F	9	impetigo with autosensitization
3	neg	neg	F	52	SLE
4	neg	neg	F	17	Livedo vasculopathy
5	neg	neg	F	42	Ischemic stroke, TIA, or other brain ischemia in patients under 50 years
6	neg	neg	F	44	Infertile
7	neg	neg	F	58	SLE
8	neg	neg	F	45	Infertile
9	pos	pos	F	47	Ischemic stroke, TIA, or other brain ischemia in patients under 50 years
10	neg	neg	M	15	SLE
11	neg	neg	F	47	Ischemic stroke, TIA, or other brain ischemia in patients under 50 years
12	neg	neg	F	35	Infertile
13	neg	neg	F	39	SLE
14	pos	neg	F	63	SLE
15	neg	neg	F	35	Infertile
16	neg	neg	F	23	SLE
17	neg	neg	F	29	SLE
18	neg	neg	F	38	Ischemic stroke, TIA, or other brain ischemia in patients under 50 years
19	neg	neg	M	23	ITP
20	neg	neg	F	45	Unprovoked VTE in those under 50 years
21	pos	pos	F	61	APS
22	pos	neg	F	51	SLE
23	neg	neg	F	35	Infertile
24	neg	neg	F	26	pyodema gangrenosum
25	neg	neg	F	89	Ischemic stroke, TIA in patients over 50 years
26	neg	neg	F	31	SLE
27	neg	neg	M	55	VTE (age >50)
28	neg	neg	F	37	SLE
29	neg	neg	F	35	prolonged fever of unknown origin
30	pos	neg	F	63	SLE
31	neg	neg	F	45	Ischemic stroke, TIA, or other brain ischemia in patients under 50 years
32	neg	neg	F	36	Infertile
33	neg	neg	F	37	Infertile
34	pos	pos	M	37	Unprovoked VTE in those under 50 years
35	neg	neg	F	18	TTP
36	neg	neg	F	31	Unprovoked VTE in those under 50 years
37	neg	neg	F	40	Recurrent Pregnancy Loss
38	neg	neg	M	26	VTE in unusual sites
39	neg	neg	F	29	Superficial vein thrombosis
40	neg	neg	F	43	Ischemic stroke, TIA, or other brain ischemia in patients under 50 years
41	neg	neg	F	36	Ischemic stroke, TIA, or other brain ischemia in patients under 50 years
42	neg	neg	F	44	Ischemic stroke, TIA, or other brain ischemia in patients under 50 years
43	neg	neg	F	59	Ischemic stroke, TIA, or other brain ischemia in patients under 50 years
44	neg	neg	F	21	SLE
45	pos	neg	F	31	SLE
46	neg	neg	F	60	SLE
47	neg	neg	F	40	Infertile
48	neg	neg	F	17	Livedo vasculopathy

No.	anti-cardiolipin	anti-beta2 glyclo protein I	Sex	Age	indications for testing
49	neg	neg	F	65	Rheumatoid arthitis
50	neg	neg	F	44	Infertile
51	neg	neg	F	72	pyodema gangrenosum
52	neg	neg	M	14	SLE
53	neg	neg	F	28	SLE
54	neg	neg	F	37	SLE
55	neg	neg	F	17	SLE
56	neg	neg	F	50	Ischemic stroke, TIA in patients over 50 years
57	neg	neg	M	54	Ischemic stroke, TIA in patients over 50 years
58	neg	neg	M	79	Ischemic stroke, TIA in patients over 50 years
59	neg	neg	F	48	Ischemic stroke, TIA, or other brain ischemia in patients under 50 years
60	neg	neg	F	42	VTE in unusual sites
61	neg	neg	F	41	Livedo vasculopathy
62	neg	neg	F	33	recurrent pregnancy loss
63	neg	neg	F	35	recurrent pregnancy loss
64	neg	neg	F	17	SLE
65	neg	neg	F	36	Infertile
66	neg	neg	F	36	Infertile
67	neg	neg	F	22	SLE
68	neg	neg	F	43	recurrent pregnancy loss
69	neg	neg	F	32	Ischemic stroke, TIA, or other brain ischemia in patients under 50 years
70	neg	neg	M	47	Ischemic stroke, TIA, or other brain ischemia in patients under 50 years
71	neg	neg	F	42	SLE
72	neg	neg	M	86	Ischemic stroke, TIA in patients over 50 years
73	neg	pos	F	42	Infertile
74	neg	neg	F	40	Ischemic stroke, TIA, or other brain ischemia in patients under 50 years
75	neg	neg	M	69	VTE in unusual sites
76	neg	neg	F	36	Infertile
77	neg	neg	F	36	Infertile
78	neg	neg	F	31	HELLP
79	neg	neg	F	34	Infertile
80	neg	neg	M	43	Ischemic stroke, TIA, or other brain ischemia in patients under 50 years
81	neg	neg	F	67	SLE
82	neg	neg	F	32	Ischemic stroke, TIA, or other brain ischemia in patients under 50 years
83	neg	neg	F	33	SLE
84	neg	neg	F	42	SLE
85	neg	neg	F	52	SLE
86	neg	neg	F	43	Infertile
87	pos	pos	F	43	recurrent pregnancy loss
88	neg	neg	F	44	recurrent pregnancy loss
89	neg	neg	F	32	endometriosis
90	neg	neg	F	48	Ischemic stroke, TIA, or other brain ischemia in patients under 50 years
91	neg	neg	F	28	Ischemic stroke, TIA, or other brain ischemia in patients under 50 years
92	neg	neg	F	31	unspecified autoimmune disease
93	neg	neg	F	63	Rheumatoid arthitis
94	neg	neg	F	20	SLE
95	neg	neg	F	40	Infertile
96	neg	neg	F	35	intravascular lymphoma

No.	anti-cardiolipin	anti-beta2 glyclo protein I	Sex	Age	indications for testing
97	neg	neg	F	46	Ischemic stroke, TIA, or other brain ischemia in patients under 50 years
98	neg	neg	F	38	Infertile
99	neg	neg	M	64	VTE (age >50) + malignancy
100	neg	neg	F	35	Infertile
101	neg	neg	M	61	Ischemic stroke, TIA in patients over 50 years
102	neg	neg	F	38	recurrent pregnancy loss
103	neg	neg	F	58	dystonia
104	neg	neg	F	41	Infertile
105	neg	neg	F	49	VTE in unusual sites
106	neg	neg	M	49	Ischemic stroke, TIA, or other brain ischemia in patients under 50 years
107	neg	neg	F	34	Infertile
108	neg	neg	F	24	Ischemic stroke, TIA, or other brain ischemia in patients under 50 years
109	neg	neg	F	44	SLE
110	neg	neg	M	42	Ischemic stroke, TIA, or other brain ischemia in patients under 50 years
111	neg	pos	F	17	SLE
112	neg	neg	F	51	Portal vein thrombosis in cirrhosis
113	neg	neg	F	42	SLE
114	neg	neg	F	37	Ischemic stroke, TIA, or other brain ischemia in patients under 50 years
115	neg	neg	F	51	VTE in unusual sites
116	neg	pos	F	17	Cutaneous Polyarteritis Nodosa
117	neg	neg	F	26	SLE
118	pos	pos	F	39	SLE
119	pos	pos	F	71	SLE
120	neg	neg	F	36	Unprovoked VTE in those under 50 years
121	neg	neg	F	55	Local arterosclerosis
122	neg	neg	F	53	Ischemic stroke, TIA in patients over 50 years
123	neg	neg	F	45	Unprovoked VTE in those under 50 years
124	neg	neg	F	37	Infertile
125	neg	neg	F	22	Recurrent Pregnancy Loss
126	neg	neg	F	43	Recurrent Pregnancy Loss
127	neg	neg	M	43	Ischemic stroke, TIA, or other brain ischemia in patients under 50 years
128	neg	neg	F	11	SLE
129	neg	neg	F	21	SLE
130	neg	neg	F	59	APS
131	neg	neg	F	20	Ischemic stroke, TIA, or other brain ischemia in patients under 50 years
132	neg	neg	F	33	Ischemic stroke, TIA, or other brain ischemia in patients under 50 years
133	neg	neg	M	25	Arterial thrombosis in younger patients
134	neg	neg	F	52	MCTD
135	neg	neg	F	40	Infertile

percentile cutoff 97.5th

	SCT				dRVVT-HemosIL				dRVVT-Hemoclot				APTT-based																		
No.	screen (sec.)	confirm (sec.)	ratio	result	screen (sec.)	confirm (sec.)	ratio	result	screen (sec.)	confirm (sec.)	ratio	result	screen (sec.)	screen ratio	result	confirm (sec.)	confirm ratio	result	screen/ confirm ratio	result	Mixing screen (sec.)	NC/ C	Mixing ratio screen	result	Mixing confirm (sec.)	NC/ C	Mixing ratio confirm	result	Mixing screen/ confirm ratio	result	ISTH method
1	43.4	37.8	1.04	NEG	45.3	31.4	1.17	POS	55.3	37.6	1.27	POS	41.3	1.06	normal	35.3	1.06	normal	1.01	NEG	39	C	1.04	NEG	33	C	1.027	NEG	1.009	NEG	NEG
2	43	33.4	1.16	POS	54.6	30.9	1.44	POS	50.3	45.3	0.96	NEG	46.1	1.19	normal	43.2	1.29	Prolong	0.92	NEG	41.1	C	1.09	NEG	36.9	C	1.148	POS	0.951	NEG	NEG
3	47.9	35.6	1.22	POS	34.1	28.9	0.96	NEG	46.3	33.7	1.19	NEG	40.6	1.04	normal	33.1	0.99	normal	1.05	NEG	39.5	C	1.05	NEG	32.3	C	1.005	NEG	1.044	NEG	NEG
4	45.4	27.8	1.48	POS	44.5	28	1.29	POS	78.7	42	1.62	POS	54.1	1.39	Prolong	36.8	1.10	normal	1.26	POS	42.4	NC	1.13	POS	33	C	1.027	NEG	1.097	POS	POS
5	44.1	38.5	1.04	NEG	49.7	32.6	1.24	POS	54.3	41.8	1.13	NEG	44.7	1.15	normal	36.6	1.10	normal	1.05	NEG	39.4	C	1.05	NEG	33.1	C	1.03	NEG	1.016	NEG	NEG
6	47.4	41.7	1.14	NEG	46.4	31.4	1.27	POS	55.7	36.1	1.34	POS	36.6	0.94	normal	31.7	0.95	normal	0.99	NEG	36.7	C	0.97	NEG	31.6	C	0.983	NEG	0.991	NEG	NEG
7	64	33.7	1.72	POS	46	31.1	1.2	POS	49.8	33.9	1.27	POS	54.2	1.39	Prolong	32	0.96	normal	1.46	POS	46	NC	1.22	POS	31.4	C	0.977	NEG	1.25	POS	POS
8	61.3	40.2	1.38	POS	35.2	29	0.99	NEG	40.1	31.6	1.10	NEG	45.2	1.16	normal	31	0.93	normal	1.25	POS	39.8	C	1.06	NEG	31	C	0.965	NEG	1.096	POS	NEG
9	119	39.8	2.7	POS	88.9	33.2	2.18	POS	115	37	2.69	POS	73.7	1.90	Prolong	31.1	0.93	normal	2.04	POS	69.2	NC	1.84	POS	32.5	C	1.011	NEG	1.817	POS	POS
10	122	83.6	1.32	POS	99.3	43.2	1.87	POS	176	65.8	2.32	POS	113.6	2.92	Prolong	69.8	2.09	Prolong	1.40	POS	133.3	NC	3.54	POS	50.7	NC	1.577	POS	2.244	POS	POS
11	80.4	46.3	1.57	POS	42.2	32.6	1.05	NEG	36.9	35.5	0.90	NEG	49.5	1.27	Prolong	33.7	1.01	normal	1.26	POS	41	C	1.09	NEG	29.6	C	0.921	NEG	1.182	POS	POS
12	46.3	35.3	1.19	POS	41.2	29.8	1.12	NEG	52.3	32.8	1.38	POS	33.8	0.87	normal	29.2	0.87	normal	0.99	NEG	34.8	C	0.92	NEG	30.1	C	0.937	NEG	0.987	NEG	NEG
13	28.2	36.5	0.7	NEG	45.5	29.8	1.24	POS	62.7	34.4	1.58	POS	43.1	1.11	normal	34	1.02	normal	1.09	NEG	42.5	NC	1.13	POS	33.4	C	1.039	NEG	1.086	POS	NEG
14	55.8	36.3	1.39	POS	38.2	28.7	1.08	NEG	33.6	31.5	0.92	NEG	51.2	1.32	Prolong	34.7	1.04	normal	1.27	POS	42.3	NC	1.12	POS	32.4	C	1.008	NEG	1.114	POS	POS
15	41.2	37.1	1	NEG	49.5	29.8	1.35	POS	56.3	35.9	1.36	POS	45.6	1.17	normal	33.9	1.01	normal	1.16	NEG	41.7	C	1.11	NEG	32.6	C	1.014	NEG	1.092	POS	NEG
16	47	34.7	1.22	POS	44.9	30.9	1.18	POS	57.4	38.7	1.29	POS	50.7	1.30	Prolong	36.6	1.10	normal	1.19	POS	42.6	NC	1.13	POS	34.2	C	1.064	NEG	1.063	NEG	POS
17	60.5	37.4	1.46	POS	38.9	27.8	1.14	NEG	49.6	29.7	1.45	POS	46.6	1.20	normal	28.1	0.84	normal	1.43	POS	38.2	C	1.01	NEG	29.5	C	0.918	NEG	1.105	POS	NEG
18	42	30.8	1.27	POS	32.3	25.7	1.02	NEG	35.8	31	1.00	NEG	35.2	0.91	normal	32	0.96	normal	0.95	NEG	36.5	C	0.97	NEG	32.1	C	0.999	NEG	0.97	NEG	NEG
19	81.2	48.4	1.52	POS	37.9	30.3	1.02	NEG	44.9	34.9	1.12	NEG	54.3	1.40	Prolong	43.3	1.30	Prolong	1.08	NEG	44.4	NC	1.18	POS	36.8	C	1.145	POS	1.03	NEG	NEG
20	61.1	42.9	1.29	POS	59.7	32.2	1.51	POS	51.8	35.4	1.27	POS	47.9	1.23	normal	35.2	1.05	normal	1.17	POS	40.5	C	1.08	NEG	32.1	C	0.999	NEG	1.077	NEG	NEG
21	157	51.9	2.73	POS	94.1	32.3	2.37	POS	90.8	35.4	2.22	POS	80.2	2.06	Prolong	38.5	1.15	normal	1.79	POS	73	NC	1.94	POS	35.2	C	1.095	NEG	1.77	POS	POS
22	71.3	45.2	1.43	POS	67.5	32.1	1.71	POS	64.3	35.2	1.58	POS	71.4	1.84	Prolong	38.4	1.15	normal	1.60	POS	62.7	NC	1.66	POS	35.2	C	1.095	NEG	1.52	POS	POS
23	41.2	37.1	1	NEG	49.5	29.8	1.35	POS	56.8	35.9	1.37	POS	45.4	1.17	normal	34.5	1.03	normal	1.13	NEG	41.6	C	1.10	NEG	32.4	C	1.008	NEG	1.096	POS	NEG
24	59.2	46.1	1.16	POS	53	34.7	1.24	POS	56	41.5	1.17	NEG	50.6	1.30	Prolong	42	1.26	Prolong	1.04	NEG	40.6	C	1.08	NEG	29.6	C	0.921	NEG	1.171	POS	NEG
25	71.1	39	1.65	POS	54.4	33.2	1.33	POS	45.8	38.5	1.03	NEG	48.2	1.24	normal	30.8	0.92	normal	1.35	POS	40.2	C	1.07	NEG	29.5	C	0.918	NEG	1.163	POS	NEG
26	80.4	47.1	1.54	POS	35.2	27.9	1.03	NEG	37.7	31.2	1.05	NEG	85.6	2.20	Prolong	39.8	1.19	normal	1.85	POS	65.1	NC	1.73	POS	34.4	C	1.07	NEG	1.615	POS	POS
27	41.4	46.5	0.9	NEG	46.2	31.8	1.24	POS	38.1	37.3	0.89	NEG	39.8	1.02	normal	34.2	1.02	normal	1.00	NEG	37.8	C	1.00	NEG	32	C	0.996	NEG	1.008	NEG	NEG
28	64.8	34.7	1.88	POS	56	31.9	1.5	POS	83.1	36.2	1.99	POS	72.6	1.87	Prolong	35.5	1.06	normal	1.76	POS	60.8	NC	1.61	POS	33	C	1.027	NEG	1.572	POS	POS
29	50.8	40.3	1.27	POS	41.6	31.4	1.14	NEG	46.3	35.3	1.14	NEG	37.2	0.96	normal	29.2	0.87	normal	1.09	NEG	37	C	0.98	NEG	29.7	C	0.924	NEG	1.063	NEG	NEG
30	65	42.6	1.54	POS	41.4	31.8	1.12	NEG	62	42.3	1.27	POS	70.6	1.82	Prolong	46.3	1.39	Prolong	1.31	POS	50	NC	1.33	POS	37.2	NC	1.157	POS	1.147	POS	POS
31	146	45.2	3.25	POS	81.5	35.7	1.96	POS	115	40.3	2.48	POS	75.7	1.95	Prolong	31.8	0.95	normal	2.05	POS	69.5	NC	1.85	POS	31.9	C	0.993	NEG	1.859	POS	POS
32	30.7	31.1	0.99	NEG	40.7	29.4	1.19	POS	49.4	33.5	1.28	POS	35	0.90	normal	29.9	0.90	normal	1.01	NEG	35.9	C	0.95	NEG	30.1	C	0.937	NEG	1.018	NEG	NEG
33	28.5	32.5	0.88	NEG	46.5	31.4	1.27	POS	50.1	33.8	1.28	POS	41.9	1.08	normal	29.3	0.88	normal	1.23	POS	36.6	C	0.97	NEG	30.7	C	0.955	NEG	1.017	NEG	NEG
34	50.3	52.7	0.95	NEG	99	33.3	2.42	POS	168	42.4	3.43	POS	137.7	3.54	Prolong	44.2	1.32	Prolong	2.68	POS	104.6	NC	2.78	POS	37.6	NC	1.17	POS	2.374	POS	POS
35	36.9	31.2	1.07	NEG	45.3	31.4	1.17	POS	53.4	38.5	1.20	NEG	38.3	0.99	normal	30.4	0.91	normal	1.08	NEG	36.2	C	0.96	NEG	29.5	C	0.918	NEG	1.047	NEG	NEG
36	38.3	41.4	0.93	NEG	47.2	31.4	1.29	POS	52.2	38.3	1.18	NEG	46.5	1.20	normal	38.3	1.15	normal	1.04	NEG	47.7	NC	1.27	POS	35	C	1.089	NEG	1.163	POS	NEG
37	56.1	46	1.23	POS	36.8	29.4	1.07	NEG	40.2	32.1	1.09	NEG	49.7	1.28	Prolong	31.4	0.94	normal	1.36	POS	43.7	NC	1.16	POS	30.3	C	0.943	NEG	1.231	POS	POS
38	38.8	57.6	0.68	NEG	74	45.7	1.39	POS	84.3	65.9	1.11	NEG	48.7	1.25	Prolong	46.5	1.39	Prolong	0.90	NEG	40.4	C	1.07	NEG	35.7	C	1.111	POS	0.966	NEG	NEG
39	43.9	40.2	1.1	NEG	43.1	31	1.19	POS	53.4	34.8	1.33	POS	43.8	1.13	normal	33	0.99	normal	1.14	NEG	39.6	C	1.05	NEG	31.4	C	0.977	NEG	1.076	NEG	NEG
40	23.7	29.2	0.82	NEG	42.9	27.6	1.33	POS	48.7	35.3	1.20	NEG	30	0.77	normal	25.1	0.75	normal	1.03	NEG	31.3	C	0.83	NEG	26.7	C	0.831	NEG	1	NEG	NEG
41	23.8	30.1	0.8	NEG	37.1	26	1.22	POS	40.8	31.1	1.14	NEG	36.7	0.94	normal	27.8	0.83	normal	1.13	NEG	33.2	C	0.88	NEG	28.4	C	0.884	NEG	0.998	NEG	NEG
42	51.3	44	1.05	NEG	44.4	30.2	1.2	POS	45.1	36.7	1.07	NEG	58	1.49	Prolong	39.7	1.19	normal	1.26	POS	49.4	NC	1.31	POS	34.6	C	1.077	NEG	1.218	POS	POS
43	23.3	28.7	0.82	NEG	44.1	27.9	1.35	POS	155	39.8	3.37	POS	108.6	2.79	Prolong	36.5	1.09	normal	2.56	POS	107.4	NC	2.85	POS	36.4	C	1.133	POS	2.518	POS	POS
44	47.6	42.8	1.12	NEG	57.4	35.3	1.39	POS	109	47	2.01	POS	40.2	1.03	normal	37	1.11	normal	0.93	NEG	37.7	C	1.00	NEG	33	C	1.027	NEG	0.975	NEG	NEG
45	76.1	52.3	1.32	POS	48.9	34.9	1.14	NEG	45.9	38.5	1.03	NEG	48.5	1.25	normal	37	1.11	normal	1.13	NEG	42.9	NC	1.14	POS	33.5	C	1.042	NEG	1.093	POS	NEG
46	141	59.4	2.14	POS	68.5	34.9	1.6	POS	84	39.6	1.84	POS	94.8	2.44	Prolong	47.9	1.43	Prolong	1.70	POS	80.6	NC	2.14	POS	37	NC	1.151	POS	1.859	POS	POS
47	40.7	39.4	1.04	NEG	31.6	28.1	0.96	NEG	38.6	29.7	1.13	NEG	35.4	0.91	normal	29.8	0.89	normal	1.02	NEG	35.7	C	0.95	NEG	30	C	0.933	NEG	1.016	NEG	NEG
48	32.1	36.9	0.88	NEG	36.9	29.9	1.06	NEG	43.7	33.4	1.13	NEG	36.3	0.93	normal	29.1	0.87	normal	1.07	NEG	36.2	C	0.96	NEG	29.9	C	0.93	NEG	1.033	NEG	NEG
49	29.4	37	0.8	NEG	29.4	28.5	0.88	NEG	28	31.5	0.77	NEG	33	0.85	normal	31.5	0.94	normal	0.90	NEG	34.4	C	0.91	NEG	30.9	C	0.961	NEG	0.95	NEG	NEG
50	37.2	36	1.04	NEG	31.1	27.1	0.98	NEG	39.1	29.7	1.14	NEG	35.7	0.92	normal	31.6	0.95	normal	0.97	NEG	36	C	0.96	NEG	31.2	C	0.971	NEG	0.985	NEG	NEG
51	25.6	24.7	1.04	NEG	34.2	28.7	1.02	NEG	42.7	31.9	1.16	NEG	28.8	0.74	normal	22.7	0.68	normal	1.09	NEG	30.7	C	0.82	NEG	25.4	C	0.79	NEG	1.032	NEG	NEG

percentile cutoff 97.5th

	SCT				dRVVT-HemosIL				dRVVT-Hemoclot				APTT-based																			
No.	screen (sec.)	confirm (sec.)	ratio	result	screen (sec.)	confirm (sec.)	ratio	result	screen (sec.)	confirm (sec.)	ratio	result	screen (sec.)	screen ratio	result	confirm (sec.)	confirm ratio	result	screen/ confirm ratio	result	Mixing screen (sec.)	NC/ C	Mixing ratio screen	result	Mixing confirm (sec.)	NC/ C	Mixing ratio confirm	result	Mixing screen/ confirm ratio	result	ISTH method
52	31.6	51	0.62	NEG	31.6	30.7	0.88	NEG	33.5	31.5	0.92	NEG	36.2	0.93	normal	37.2	1.11	normal	0.84	NEG	37.7	C	1.00	NEG	33.8	C	1.052	NEG	0.952	NEG	NEG
53	38.4	36.8	1.05	NEG	33.7	30.2	0.96	NEG	37.6	32.2	1.01	NEG	37	0.95	normal	30.2	0.90	normal	1.05	NEG	36.6	C	0.97	NEG	30.4	C	0.946	NEG	1.027	NEG	NEG
54	34.8	39.7	0.91	NEG	30.1	28.7	0.9	NEG	32.1	29	0.96	NEG	36.8	0.95	normal	32.7	0.98	normal	0.97	NEG	37.5	C	1.00	NEG	32.3	C	1.005	NEG	0.991	NEG	NEG
55	39.6	39.7	1	NEG	39.1	29.3	1.14	NEG	37	31	1.03	NEG	39.3	1.01	normal	33	0.99	normal	1.02	NEG	37.7	C	1.00	NEG	31.2	C	0.971	NEG	1.031	NEG	NEG
56	50	50.4	1	NEG	35	31.7	0.95	NEG	39.6	36.3	0.95	NEG	38.6	0.99	normal	33.8	1.01	normal	0.98	NEG	37.7	C	1.00	NEG	32.4	C	1.008	NEG	0.993	NEG	NEG
57	32.8	34.3	0.96	NEG	29.2	28.7	0.87	NEG	32.2	31.1	0.90	NEG	37.1	0.95	normal	29.5	0.88	normal	1.08	NEG	35.6	C	0.95	NEG	29.7	C	0.924	NEG	1.023	NEG	NEG
58	34.6	44	0.79	NEG	40.7	34.7	1	NEG	41	38.6	0.92	NEG	37.9	0.98	normal	33.3	1.00	normal	0.98	NEG	36.4	C	0.97	NEG	31.8	C	0.989	NEG	0.977	NEG	NEG
59	32.3	35.1	0.93	NEG	35.9	30	1.03	NEG	39.4	35.7	0.96	NEG	35.8	0.92	normal	30.6	0.92	normal	1.01	NEG	36.7	C	0.97	NEG	30.7	C	0.955	NEG	1.02	NEG	NEG
60	38.9	40.1	0.98	NEG	31.2	26.8	1	NEG	40.3	29.6	1.18	NEG	38.7	1.00	normal	33	0.99	normal	1.01	NEG	37.9	C	1.01	NEG	32.4	C	1.008	NEG	0.998	NEG	NEG
61	27.9	35.8	0.78	NEG	32.4	27.3	1.02	NEG	41	30.3	1.17	NEG	28.6	0.74	normal	27.5	0.82	normal	0.89	NEG	30.5	C	0.81	NEG	28.1	C	0.874	NEG	0.926	NEG	NEG
62	43.2	41.1	1.06	NEG	42.6	32.2	1.13	NEG	45.3	35.3	1.11	NEG	41.3	1.06	normal	35.6	1.07	normal	1.00	NEG	39.6	C	1.05	NEG	33.5	C	1.042	NEG	1.009	NEG	NEG
63	50.6	48.7	1.05	NEG	36.7	30.3	1.04	NEG	43	34.4	1.08	NEG	42.5	1.09	normal	37	1.11	normal	0.99	NEG	39.1	C	1.04	NEG	33.7	C	1.049	NEG	0.99	NEG	NEG
64	25.1	37.6	0.67	NEG	29.3	27.8	0.9	NEG	40.3	30.2	1.16	NEG	26.6	0.68	normal	26.3	0.79	normal	0.87	NEG	28.8	C	0.76	NEG	27.3	C	0.849	NEG	0.9	NEG	NEG
65	35.6	38.4	0.93	NEG	26	26.5	0.84	NEG	27.5	28.3	0.84	NEG	39.8	1.02	normal	34.9	1.04	normal	0.98	NEG	38.4	C	1.02	NEG	33.3	C	1.036	NEG	0.984	NEG	NEG
66	36.3	35.2	1.04	NEG	34.3	27.9	1.05	NEG	45.3	32.9	1.19	NEG	35.2	0.91	normal	31.3	0.94	normal	0.97	NEG	36.4	C	0.97	NEG	31.3	C	0.974	NEG	0.992	NEG	NEG
67	28.8	35.2	0.82	NEG	30.8	26.8	0.98	NEG	37.7	31.2	1.05	NEG	32.5	0.84	normal	27.9	0.84	normal	1.00	NEG	34.2	C	0.91	NEG	28.8	C	0.896	NEG	1.013	NEG	NEG
68	43.5	43.2	1.01	NEG	34.6	30.2	0.98	NEG	39.1	32.8	1.03	NEG	42.3	1.09	normal	37.4	1.12	normal	0.97	NEG	39	C	1.04	NEG	33.6	C	1.045	NEG	0.991	NEG	NEG
69	32.6	34	0.97	NEG	33	29.4	0.96	NEG	37.7	33.7	0.97	NEG	33.6	0.86	normal	29.4	0.88	normal	0.98	NEG	34.6	C	0.92	NEG	29.7	C	0.924	NEG	0.994	NEG	NEG
70	42.3	42.1	1.01	NEG	35.4	37.8	1.09	NEG	40.9	35.2	1.01	NEG	44.2	1.14	normal	38.3	1.15	normal	0.99	NEG	39.5	C	1.05	NEG	33.7	C	1.049	NEG	1	NEG	NEG
71	39.5	67.8	0.59	NEG	35.5	33.6	0.91	NEG	35.7	37.2	0.83	NEG	41.5	1.07	normal	43.4	1.30	Prolong	0.82	NEG	37.8	C	1.00	NEG	35.2	C	1.095	NEG	0.916	NEG	NEG
72	38.1	38.7	0.99	NEG	31.8	28.3	0.96	NEG	41	33.8	1.05	NEG	44.7	1.15	normal	34.4	1.03	normal	1.12	NEG	38.7	C	1.03	NEG	32.8	C	1.021	NEG	1.007	NEG	NEG
73	51.9	47.5	1.1	NEG	46.2	35.3	1.12	NEG	59	42	1.22	NEG	44.1	1.13	normal	38.5	1.15	normal	0.98	NEG	40.4	C	1.07	NEG	34.4	C	1.07	NEG	1.002	NEG	NEG
74	38.8	38.2	1.02	NEG	40.3	30.9	1.12	NEG	46.1	37.2	1.07	NEG	36.7	0.94	normal	33.7	1.01	normal	0.94	NEG	37.2	C	0.99	NEG	32.5	C	1.011	NEG	0.977	NEG	NEG
75	41.3	39.8	1.04	NEG	40.8	31.2	1.12	NEG	38.7	32.2	1.04	NEG	38.4	0.99	normal	33.1	0.99	normal	1.00	NEG	37	C	0.98	NEG	31.6	C	0.983	NEG	0.999	NEG	NEG
76	30.1	32.8	0.92	NEG	30.4	25.4	0.99	NEG	34.4	28.2	1.06	NEG	33.1	0.85	normal	30.1	0.90	normal	0.95	NEG	34.7	C	0.92	NEG	30.1	C	0.937	NEG	0.984	NEG	NEG
77	44.4	44.5	1	NEG	42.7	31.6	1.16	POS	44.8	34.8	1.12	NEG	40.8	1.05	normal	34	1.02	normal	1.03	NEG	38.5	C	1.02	NEG	32.6	C	1.014	NEG	1.008	NEG	NEG
78	45.9	42.4	1.09	NEG	34.5	30	0.99	NEG	35.7	31.3	0.99	NEG	41.1	1.06	normal	34.2	1.02	normal	1.03	NEG	37.7	C	1.00	NEG	32	C	0.996	NEG	1.005	NEG	NEG
79	31.9	33.7	0.95	NEG	34.9	28.6	1.05	NEG	41.3	32.5	1.10	NEG	35	0.90	normal	30.1	0.90	normal	1.00	NEG	35.8	C	0.95	NEG	30.4	C	0.946	NEG	1.005	NEG	NEG
80	31.8	34	0.94	NEG	27.9	26.5	0.9	NEG	32	29	0.96	NEG	32.7	0.84	normal	28.5	0.85	normal	0.99	NEG	31.5	C	0.84	NEG	28.6	C	0.89	NEG	0.94	NEG	NEG
81	34.5	51.7	0.67	NEG	38.7	33.2	1	NEG	42.1	42.1	0.87	NEG	39.4	1.01	normal	39.2	1.17	normal	0.86	NEG	37.6	C	1.00	NEG	35.1	C	1.092	NEG	0.914	NEG	NEG
82	32.6	34	0.97	NEG	33	29.4	0.96	NEG	34.2	31.4	0.94	NEG	32.5	0.84	normal	28.2	0.84	normal	0.99	NEG	33.5	C	0.89	NEG	28.9	C	0.899	NEG	0.989	NEG	NEG
83	30.9	37	0.84	NEG	31.1	28.1	0.95	NEG	36.2	29.1	1.08	NEG	30.7	0.79	normal	27.1	0.81	normal	0.97	NEG	30.9	C	0.82	NEG	26.9	C	0.837	NEG	0.98	NEG	NEG
84	46.9	39.3	1.02	NEG	40.7	29.9	1.17	POS	49	32.9	1.29	POS	42	1.08	normal	30.9	0.93	normal	1.17	POS	38.8	C	1.03	NEG	30.5	C	0.949	NEG	1.086	POS	NEG
85	40.1	46.2	0.87	NEG	34.2	30.6	0.96	NEG	37.7	32.9	0.99	NEG	34.5	0.89	normal	30.8	0.92	normal	0.96	NEG	35.9	C	0.95	NEG	30.7	C	0.955	NEG	0.998	NEG	NEG
86	39.9	37	1.09	NEG	30	27	0.95	NEG	33.9	29.2	1.01	NEG	37.2	0.96	normal	32.2	0.96	normal	0.99	NEG	37.2	C	0.99	NEG	31.6	C	0.983	NEG	1.005	NEG	NEG
87	40.8	40.8	1.01	NEG	36.8	28.4	1.11	NEG	38.4	30.6	1.09	NEG	40.4	1.04	normal	35	1.05	normal	0.99	NEG	39.5	C	1.05	NEG	33.5	C	1.042	NEG	1.006	NEG	NEG
88	46.5	39	1.2	NEG	41.7	31.9	1.12	NEG	38.9	33.2	1.02	NEG	46.9	1.21	normal	36.4	1.09	normal	1.11	NEG	40.9	C	1.09	NEG	33	C	1.027	NEG	1.058	NEG	NEG
89	47.5	50.6	0.95	NEG	35	32.4	0.93	NEG	43.9	35.8	1.06	NEG	41.3	1.06	normal	38.3	1.15	normal	0.93	NEG	38.7	C	1.03	NEG	34.1	C	1.061	NEG	0.969	NEG	NEG
90	47	50.3	0.94	NEG	38.3	33.6	0.98	NEG	36	37.6	0.83	NEG	43.3	1.11	normal	35.7	1.07	normal	1.04	NEG	41.2	C	1.09	NEG	33.6	C	1.045	NEG	1.046	NEG	NEG
91	38.7	52.7	0.74	NEG	44.6	35.3	1.08	NEG	57.7	40.3	1.24	POS	39.8	1.02	normal	35.2	1.05	normal	0.97	NEG	39.5	C	1.05	NEG	33.8	C	1.052	NEG	0.997	NEG	NEG
92	33.3	36.2	0.93	NEG	39.2	31.9	1.05	NEG	60.6	43.5	1.21	NEG	32	0.82	normal	32.7	0.98	normal	0.84	NEG	34.5	C	0.92	NEG	30.7	C	0.955	NEG	0.959	NEG	NEG
93	45.1	47.4	0.96	NEG	43.6	35.6	1.05	NEG	46.9	40.6	1.00	NEG	37.6	0.97	normal	32.3	0.97	normal	1.00	NEG	36.9	C	0.98	NEG	30.8	C	0.958	NEG	1.022	NEG	NEG
94	36.6	37.3	0.99	NEG	38.5	28.7	1.15	NEG	48.6	34.8	1.21	NEG	38	0.98	normal	33.4	1.00	normal	0.98	NEG	37.7	C	1.00	NEG	31.8	C	0.989	NEG	1.012	NEG	NEG
95	41.5	39.5	1.06	NEG	34	27.7	1.05	NEG	43.1	28.9	1.29	POS	38.6	0.99	normal	34.1	1.02	normal	0.97	NEG	38.2	C	1.01	NEG	32.7	C	1.017	NEG	0.997	NEG	NEG
96	38	38	1.01	NEG	36.4	30	1.04	NEG	45.1	40.1	0.97	NEG	52.1	1.34	Prolong	35.4	1.06	normal	1.26	POS	44.6	NC	1.18	POS	31.9	C	0.993	NEG	1.193	POS	POS
97	26.9	30.1	0.9	NEG	30.8	29.4	0.9	NEG	30	31.7	0.82	NEG	30.3	0.78	normal	26.1	0.78	normal	1.00	NEG	32.5	C	0.86	NEG	27.7	C	0.862	NEG	1.001	NEG	NEG
98	41.3	44.3	0.94	NEG	33	31.1	0.91	NEG	32.7	32.9	0.86	NEG	37.9	0.98	normal	35.1	1.05	normal	0.93	NEG	38	C	1.01	NEG	33.2	C	1.033	NEG	0.977	NEG	NEG
99	40.4	78	0.52	NEG	39	38.6	0.87	NEG	46.9	50.6	0.80	NEG	38.8	1.00	normal	40.2	1.20	normal	0.83	NEG	36.2	C	0.96	NEG	33.3	C	1.036	NEG	0.928	NEG	NEG
100	49.2	51.1	0.97	NEG	29.2	28.2	0.89	NEG	31.4	29.3	0.93	NEG	46.8	1.20	normal	44.8	1.34	Prolong	0.90	NEG	40.7	C	1.08	NEG	36.5	C	1.136	POS	0.952	NEG	NEG
101	37	37.5	0.99	NEG	33.8	29.9	0.97	NEG	37.6	31.5	1.03	NEG	36.3	0.93	normal	28	0.84	normal	1.11	NEG	35.7	C	0.95	NEG	28.4	C	0.884	NEG	1.073	NEG	NEG
102	35.4	34.7	1.03	NEG	30.9	27.8	0.95	NEG	28.9	27.9	0.90	NEG	36.6	0.94	normal	32.1	0.96	normal	0.98	NEG	37.3	C	0.99	NEG	31.5	C	0.98	NEG	1.011	NEG	NEG

percentile cutoff 97.5th

	SCT				dRVVT-HemosIL				dRVVT-Hemoclot				APTT-based																		
No.	screen (sec.)	confirm (sec.)	ratio	result	screen (sec.)	confirm (sec.)	ratio	result	screen (sec.)	confirm (sec.)	ratio	result	screen (sec.)	screen ratio	result	confirm (sec.)	confirm ratio	result	screen/ confirm ratio	result	Mixing screen (sec.)	NC/ C	Mixing ratio screen	result	Mixing confirm (sec.)	NC/ C	Mixing ratio confirm	result	Mixing screen/ confirm ratio	result	ISTH method
103	41.7	44.3	0.95	NEG	34	30.1	0.97	NEG	44.5	34.3	1.12	NEG	38	0.98	normal	32.8	0.98	normal	1.00	NEG	37.5	C	1.00	NEG	32	C	0.996	NEG	1	NEG	NEG
104	38.6	44.2	0.88	NEG	33.2	31.7	0.9	NEG	37.4	34.1	0.95	NEG	39.1	1.01	normal	35	1.05	normal	0.96	NEG	38.6	C	1.02	NEG	33	C	1.027	NEG	0.998	NEG	NEG
105	38.7	40.5	0.96	NEG	32	30.9	0.89	NEG	36.4	34.3	0.92	NEG	36.5	0.94	normal	33.1	0.99	normal	0.95	NEG	37	C	0.98	NEG	32.8	C	1.021	NEG	0.963	NEG	NEG
106	38.7	36	1.08	NEG	36.2	28.6	1.08	NEG	41	32.1	1.11	NEG	39.9	1.03	normal	31.4	0.94	normal	1.09	NEG	38.5	C	1.02	NEG	30.9	C	0.961	NEG	1.063	NEG	NEG
107	49.2	51.1	0.97	NEG	29.2	28.2	0.89	NEG	32.8	29.9	0.95	NEG	47	1.21	normal	43.8	1.31	Prolong	0.92	NEG	40.6	C	1.08	NEG	36.5	C	1.136	POS	0.949	NEG	NEG
108	41.1	46.9	0.88	NEG	35.5	30.8	0.99	NEG	37.4	35.3	0.92	NEG	40.3	1.04	normal	35.6	1.07	normal	0.97	NEG	39.2	C	1.04	NEG	33.5	C	1.042	NEG	0.999	NEG	NEG
109	51.8	49.7	1.05	NEG	46.3	33.6	1.18	POS	79.8	47.9	1.44	POS	54.6	1.41	Prolong	43.4	1.30	Prolong	1.08	NEG	44.9	NC	1.19	POS	36.3	C	1.129	POS	1.056	NEG	NEG
110	38.5	38.8	1	NEG	40.7	32	1.09	NEG	42.8	36.8	1.01	NEG	36.2	0.93	normal	31.7	0.95	normal	0.98	NEG	35.9	C	0.95	NEG	30.3	C	0.943	NEG	1.011	NEG	NEG
111	93.6	39.7	2.37	POS	125.6	35.6	3.02	POS	240	56	3.71	POS	124.2	3.20	Prolong	50.5	1.51	Prolong	2.11	POS	95.7	NC	2.54	POS	40.2	NC	1.251	POS	2.032	POS	POS
112	42.9	54.4	0.8	NEG	64.6	66	0.84	NEG	74.6	115.7	0.56	NEG	57.1	1.47	Prolong	55.9	1.67	Prolong	0.88	NEG	32.8	C	0.87	NEG	32.5	C	1.011	NEG	0.861	NEG	NEG
113	54.8	39.6	1.39	POS	48.1	32.4	1.27	POS	53.8	36.8	1.27	POS	45.7	1.18	normal	29.5	0.88	normal	1.33	POS	40.2	C	1.07	NEG	29.7	C	0.924	NEG	1.155	POS	NEG
114	51	46.4	1.11	NEG	41.6	30.8	1.16	POS	38	34.2	0.96	NEG	43.3	1.11	normal	34.1	1.02	normal	1.09	NEG	39.1	C	1.04	NEG	32.4	C	1.008	NEG	1.03	NEG	NEG
115	42.5	40.9	1.05	NEG	36.4	26.3	1.19	POS	35.3	29	1.06	NEG	38.4	0.99	normal	33.7	1.01	normal	0.98	NEG	37.6	C	1.00	NEG	31.7	C	0.986	NEG	1.012	NEG	NEG
116	56.5	42.7	1.33	POS	47.2	33.6	1.2	POS	39.2	35.9	0.95	NEG	50.6	1.30	Prolong	36.2	1.08	normal	1.20	POS	43.7	NC	1.16	POS	33.8	C	1.052	NEG	1.103	POS	POS
117	94.1	48.3	1.96	POS	50.6	30.5	1.42	POS	68.7	41.6	1.43	POS	76.7	1.97	Prolong	51.6	1.54	Prolong	1.28	POS	65	NC	1.73	POS	38.2	NC	1.189	POS	1.452	POS	POS
118	141	44.1	3.21	POS	75.9	30.7	2.12	POS	78.2	32.6	2.08	POS	98.8	2.54	Prolong	32.7	0.98	normal	2.60	POS	75.5	NC	2.00	POS	31.6	C	0.983	NEG	2.039	POS	POS
119	237	47.3	5.04	POS	114.6	33	2.98	POS	134	42.1	2.75	POS	120.2	3.09	Prolong	44.1	1.32	Prolong	2.34	POS	119.9	NC	3.18	POS	43	NC	1.338	POS	2.38	POS	POS
120	131	40.7	3.23	POS	60	28.4	1.81	POS	106	33.3	2.75	POS	95.6	2.46	Prolong	42.6	1.28	Prolong	1.93	POS	77.8	NC	2.07	POS	35.7	C	1.111	POS	1.86	POS	POS
121	39.1	39.8	0.99	NEG	29.6	29.5	0.86	NEG	38.4	33.2	1.00	NEG	35.3	0.91	normal	30.6	0.92	normal	0.99	NEG	36.8	C	0.98	NEG	30.9	C	0.961	NEG	1.016	NEG	NEG
122	48.6	41.2	1.19	NEG	34.8	31.1	0.96	NEG	41.7	35.1	1.03	NEG	42	1.08	normal	33.5	1.00	normal	1.08	NEG	39.1	C	1.04	NEG	32.3	C	1.005	NEG	1.033	NEG	NEG
123	47.4	47.9	1	NEG	37	29.9	1.06	NEG	32.8	31	0.92	NEG	40	1.03	normal	33.2	0.99	normal	1.04	NEG	39	C	1.04	NEG	32.2	C	1.002	NEG	1.034	NEG	NEG
124	37.9	37.8	1.01	NEG	27.6	26.7	0.89	NEG	30.1	28.4	0.92	NEG	37.1	0.95	normal	33.2	0.99	normal	0.96	NEG	37.1	C	0.99	NEG	31.7	C	0.986	NEG	0.999	NEG	NEG
125	36.1	39.2	0.93	NEG	32.5	28.2	0.99	NEG	32.8	29.9	0.95	NEG	41.9	1.08	normal	35	1.05	normal	1.03	NEG	39.6	C	1.05	NEG	33	C	1.027	NEG	1.024	NEG	NEG
126	48.9	56	0.88	NEG	35	31.8	0.94	NEG	36.3	35.6	0.88	NEG	44	1.13	normal	39.7	1.19	normal	0.95	NEG	39.9	C	1.06	NEG	34.8	C	1.083	NEG	0.978	NEG	NEG
127	35.2	37.5	0.95	NEG	31.5	45.4	0.59	NEG	39.3	30.6	1.11	NEG	33.8	0.87	normal	28	0.84	normal	1.04	NEG	34.9	C	0.93	NEG	28.6	C	0.89	NEG	1.041	NEG	NEG
128	60.1	65.7	0.92	NEG	44.9	33.3	1.16	POS	41.4	38.2	0.94	NEG	61.2	1.57	Prolong	46	1.38	Prolong	1.14	NEG	47.8	NC	1.27	POS	37.6	NC	1.17	POS	1.085	POS	NEG
129	52.3	44.1	1.19	NEG	47.2	30.3	1.33	POS	36.5	29.2	1.08	NEG	43.8	1.13	normal	32.2	0.96	normal	1.17	POS	41.1	C	1.09	NEG	31.7	C	0.986	NEG	1.106	POS	NEG
130	75	36.1	2.09	POS	57.1	31	1.58	POS	77.7	34.1	1.98	POS	94.5	2.43	Prolong	37	1.11	normal	2.20	POS	98.4	NC	2.61	POS	36.1	C	1.123	POS	2.326	POS	POS
131	42.3	48	0.89	NEG	41.2	32.4	1.09	NEG	44.9	39.2	0.99	NEG	41.7	1.07	normal	38.9	1.16	normal	0.92	NEG	40.1	C	1.06	NEG	34.6	C	1.077	NEG	0.989	NEG	NEG
132	45.4	47.2	0.97	NEG	35.4	28.9	1.05	NEG	40.7	34.6	1.02	NEG	42.9	1.10	normal	36	1.08	normal	1.02	NEG	39.3	C	1.04	NEG	33.4	C	1.039	NEG	1.004	NEG	NEG
133	30.4	29.2	1.05	NEG	31.3	25.7	1.04	NEG	31	27	1.00	NEG	30.6	0.79	normal	24.7	0.74	normal	1.06	NEG	31.8	C	0.84	NEG	26.2	C	0.815	NEG	1.036	NEG	NEG
134	49	58.6	0.84	NEG	36.6	34.3	0.91	NEG	38.9	37.1	0.91	NEG	40.8	1.05	normal	37.9	1.13	normal	0.93	NEG	39.2	C	1.04	NEG	34.3	C	1.067	NEG	0.975	NEG	NEG
135	41.3	35.3	1.18	NEG	39	29.3	1.14	NEG	24.9	24.6	0.88	NEG	32.8	0.84	normal	28.7	0.86	normal	0.98	NEG	34.5	C	0.92	NEG	29.5	C	0.918	NEG	0.998	NEG	NEG

percentile cutoff 99th

	SCT				dRVVT-HemosIL				dRVVT-Hemoclot				APTT-based																		
No.	screen (sec.)	confirm (sec.)	ratio	result	screen (sec.)	confirm (sec.)	ratio	result	screen (sec.)	confirm (sec.)	ratio	result	screen (sec.)	screen ratio	result	confirm (sec.)	confirm ratio	result	screen/ confirm ratio	result	Mixing screen (sec.)	NC/ C	Mixing ratio screen	result	Mixing confirm (sec.)	NC/ C	Mixing ratio confirm	result	Mixing screen/ confirm ratio	result	ISTH method
1	43.4	37.8	1	NEG	45.3	31.4	1.17	POS	55.3	37.6	1.27	POS	41.3	1.06	Normal	35.3	1.06	Normal	1.01	NEG	39	C	1.04	NEG	33	C	1.03	NEG	1.01	NEG	NEG
2	43	33.4	1.2	POS	54.6	30.9	1.44	POS	50.3	45.3	0.96	NEG	46.1	1.19	Normal	43.2	1.29	Prolong	0.92	NEG	41.1	C	1.09	NEG	36.9	C	1.15	NEG	0.95	NEG	NEG
3	47.9	35.6	1.2	POS	34.1	28.9	0.96	NEG	46.3	33.7	1.19	NEG	40.6	1.04	Normal	33.1	0.99	Normal	1.05	NEG	39.5	C	1.05	NEG	32.3	C	1.00	NEG	1.04	NEG	NEG
4	45.4	27.8	1.5	POS	44.5	28	1.29	POS	78.7	42	1.62	POS	54.1	1.39	Prolong	36.8	1.10	Normal	1.26	POS	42.4	C	1.13	NEG	33	C	1.03	NEG	1.10	POS	POS
5	44.1	38.5	1	NEG	49.7	32.6	1.24	POS	54.3	41.8	1.13	NEG	44.7	1.15	Normal	36.6	1.10	Normal	1.05	NEG	39.4	C	1.05	NEG	33.1	C	1.03	NEG	1.02	NEG	NEG
6	47.4	41.7	1.1	NEG	46.4	31.4	1.27	POS	55.7	36.1	1.34	POS	36.6	0.94	Normal	31.7	0.95	Normal	0.99	NEG	36.7	C	0.97	NEG	31.6	C	0.98	NEG	0.99	NEG	NEG
7	64	33.7	1.7	POS	46	31.1	1.2	POS	49.8	33.9	1.27	POS	54.2	1.39	Prolong	32	0.96	Normal	1.46	POS	46	NC	1.22	POS	31.4	C	0.98	NEG	1.25	POS	POS
8	61.3	40.2	1.4	POS	35.2	29	0.99	NEG	40.1	31.6	1.10	NEG	45.2	1.16	Normal	31	0.93	Normal	1.25	POS	39.8	C	1.06	NEG	31	C	0.96	NEG	1.10	POS	NEG
9	119	39.8	2.7	POS	88.9	33.2	2.18	POS	115	37	2.69	POS	73.7	1.90	Prolong	31.1	0.93	Normal	2.04	POS	69.2	NC	1.84	POS	32.5	C	1.01	NEG	1.82	POS	POS
10	122	83.6	1.3	POS	99.3	43.2	1.87	POS	176	65.8	2.32	POS	114	2.92	Prolong	69.8	2.09	Prolong	1.40	POS	133	NC	3.54	POS	50.7	NC	1.58	POS	2.24	POS	POS
11	80.4	46.3	1.6	POS	42.2	32.6	1.05	NEG	36.9	35.5	0.90	NEG	49.5	1.27	Prolong	33.7	1.01	Normal	1.26	POS	41	C	1.09	NEG	29.6	C	0.92	NEG	1.18	POS	POS
12	46.3	35.3	1.2	POS	41.2	29.8	1.12	NEG	52.3	32.8	1.38	POS	33.8	0.87	Normal	29.2	0.87	Normal	0.99	NEG	34.8	C	0.92	NEG	30.1	C	0.94	NEG	0.99	NEG	NEG
13	28.2	36.5	0.7	NEG	45.5	29.8	1.24	POS	62.7	34.4	1.58	POS	43.1	1.11	Normal	34	1.02	Normal	1.09	NEG	42.5	C	1.13	NEG	33.4	C	1.04	NEG	1.09	NEG	NEG
14	55.8	36.3	1.4	POS	38.2	28.7	1.08	NEG	33.6	31.5	0.92	NEG	51.2	1.32	Prolong	34.7	1.04	Normal	1.27	POS	42.3	C	1.12	NEG	32.4	C	1.01	NEG	1.11	POS	POS
15	41.2	37.1	1	NEG	49.5	29.8	1.35	POS	56.3	35.9	1.36	POS	45.6	1.17	Normal	33.9	1.01	Normal	1.16	NEG	41.7	C	1.11	NEG	32.6	C	1.01	NEG	1.09	POS	NEG
16	47	34.7	1.2	POS	44.9	30.9	1.18	POS	57.4	38.7	1.29	POS	50.7	1.30	Prolong	36.6	1.10	Normal	1.19	NEG	42.6	C	1.13	POS	34.2	C	1.06	NEG	1.06	NEG	NEG
17	60.5	37.4	1.5	POS	38.9	27.8	1.14	NEG	49.6	29.7	1.45	POS	46.6	1.20	Normal	28.1	0.84	Normal	1.43	POS	38.2	C	1.01	NEG	29.5	C	0.92	NEG	1.11	POS	NEG
18	42	30.8	1.3	POS	32.3	25.7	1.02	NEG	35.8	31	1.00	NEG	35.2	0.91	Normal	32	0.96	Normal	0.95	NEG	36.5	C	0.97	NEG	32.1	C	1.00	NEG	0.97	NEG	NEG
19	81.2	48.4	1.5	POS	37.9	30.3	1.02	NEG	44.9	34.9	1.12	NEG	54.3	1.40	Prolong	43.3	1.30	Prolong	1.08	NEG	44.4	NC	1.18	POS	36.8	C	1.14	NEG	1.03	NEG	NEG
20	61.1	42.9	1.3	POS	59.7	32.2	1.51	POS	51.8	35.4	1.27	POS	47.9	1.23	Normal	35.2	1.05	Normal	1.17	NEG	40.5	C	1.08	NEG	32.1	C	1.00	NEG	1.08	NEG	NEG
21	157	51.9	2.7	POS	94.1	32.3	2.37	POS	90.8	35.4	2.22	POS	80.2	2.06	Prolong	38.5	1.15	Normal	1.79	POS	73	NC	1.94	POS	35.2	C	1.10	NEG	1.77	POS	POS
22	71.3	45.2	1.4	POS	67.5	32.1	1.71	POS	64.3	35.2	1.58	POS	71.4	1.84	Prolong	38.4	1.15	Normal	1.60	POS	62.7	NC	1.66	POS	35.2	C	1.10	NEG	1.52	POS	POS
23	41.2	37.1	1	NEG	49.5	29.8	1.35	POS	56.8	35.9	1.37	POS	45.4	1.17	Normal	34.5	1.03	Normal	1.13	NEG	41.6	C	1.10	NEG	32.4	C	1.01	NEG	1.10	POS	NEG
24	59.2	46.1	1.2	POS	53	34.7	1.24	POS	56	41.5	1.17	NEG	50.6	1.30	Prolong	42	1.26	Normal	1.04	NEG	40.6	C	1.08	NEG	29.6	C	0.92	NEG	1.17	POS	NEG
25	71.1	39	1.7	POS	54.4	33.2	1.33	POS	45.8	38.5	1.03	NEG	48.2	1.24	Normal	30.8	0.92	Normal	1.35	POS	40.2	C	1.07	NEG	29.5	C	0.92	NEG	1.16	POS	NEG
26	80.4	47.1	1.5	POS	35.2	27.9	1.03	NEG	37.7	31.2	1.05	NEG	85.6	2.20	Prolong	39.8	1.19	Normal	1.85	POS	65.1	NC	1.73	POS	34.4	C	1.07	NEG	1.62	POS	POS
27	41.4	46.5	0.9	NEG	46.2	31.8	1.24	POS	38.1	37.3	0.89	NEG	39.8	1.02	Normal	34.2	1.02	Normal	1.00	NEG	37.8	C	1.00	NEG	32	C	1.00	NEG	1.01	NEG	NEG
28	64.8	34.7	1.9	POS	56	31.9	1.5	POS	83.1	36.2	1.99	POS	72.6	1.87	Prolong	35.5	1.06	Normal	1.76	POS	60.8	NC	1.61	POS	33	C	1.03	NEG	1.57	POS	POS
29	50.8	40.3	1.3	POS	41.6	31.4	1.14	NEG	46.3	35.3	1.14	NEG	37.2	0.96	Normal	29.2	0.87	Normal	1.09	NEG	37	C	0.98	NEG	29.7	C	0.92	NEG	1.06	NEG	NEG
30	65	42.6	1.5	POS	41.4	31.8	1.12	NEG	62	42.3	1.27	POS	70.6	1.82	Prolong	46.3	1.39	Prolong	1.31	POS	50	NC	1.33	POS	37.2	NC	1.16	POS	1.15	POS	POS
31	146	45.2	3.3	POS	81.5	35.7	1.96	POS	115	40.3	2.48	POS	75.7	1.95	Prolong	31.8	0.95	Normal	2.05	POS	69.5	NC	1.85	POS	31.9	C	0.99	NEG	1.86	POS	POS
32	30.7	31.1	1	NEG	40.7	29.4	1.19	POS	49.4	33.5	1.28	POS	35	0.90	Normal	29.9	0.90	Normal	1.01	NEG	35.9	C	0.95	NEG	30.1	C	0.94	NEG	1.02	NEG	NEG
33	28.5	32.5	0.9	NEG	46.5	31.4	1.27	POS	50.1	33.8	1.28	POS	41.9	1.08	Normal	29.3	0.88	Normal	1.23	NEG	36.6	C	0.97	NEG	30.7	C	0.96	NEG	1.02	NEG	NEG
34	50.3	52.7	1	NEG	99	33.3	2.42	POS	168	42.4	3.43	POS	138	3.54	Prolong	44.2	1.32	Prolong	2.68	POS	105	NC	2.78	POS	37.6	NC	1.17	POS	2.37	POS	POS
35	36.9	31.2	1.1	NEG	45.3	31.4	1.17	POS	53.4	38.5	1.20	NEG	38.3	0.99	Normal	30.4	0.91	Normal	1.08	NEG	36.2	C	0.96	NEG	29.5	C	0.92	NEG	1.05	NEG	NEG
36	38.3	41.4	0.9	NEG	47.2	31.4	1.29	POS	52.2	38.3	1.18	NEG	46.5	1.20	Normal	38.3	1.15	Normal	1.04	NEG	47.7	NC	1.27	POS	35	C	1.09	NEG	1.16	POS	NEG
37	56.1	46	1.2	POS	36.8	29.4	1.07	NEG	40.2	32.1	1.09	NEG	49.7	1.28	Prolong	31.4	0.94	Normal	1.36	POS	43.7	NC	1.16	POS	30.3	C	0.94	NEG	1.23	POS	POS
38	38.8	57.6	0.7	NEG	74	45.7	1.39	POS	84.3	65.9	1.11	NEG	48.7	1.25	Normal	46.5	1.39	Prolong	0.90	NEG	40.4	C	1.07	NEG	35.7	C	1.11	NEG	0.97	NEG	NEG
39	43.9	40.2	1.1	NEG	43.1	31	1.19	POS	53.4	34.8	1.33	POS	43.8	1.13	Normal	33	0.99	Normal	1.14	NEG	39.6	C	1.05	NEG	31.4	C	0.98	NEG	1.08	NEG	NEG
40	23.7	29.2	0.8	NEG	42.9	27.6	1.33	POS	48.7	35.3	1.20	NEG	30	0.77	Normal	25.1	0.75	Normal	1.03	NEG	31.3	C	0.83	NEG	26.7	C	0.83	NEG	1.00	NEG	NEG
41	23.8	30.1	0.8	NEG	37.1	26	1.22	POS	40.8	31.1	1.14	NEG	36.7	0.94	Normal	27.8	0.83	Normal	1.13	NEG	33.2	C	0.88	NEG	28.4	C	0.88	NEG	1.00	NEG	NEG
42	51.3	44	1.1	NEG	44.4	30.2	1.2	POS	45.1	36.7	1.07	NEG	58	1.49	Prolong	39.7	1.19	Normal	1.26	POS	49.4	NC	1.31	POS	34.6	C	1.08	NEG	1.22	POS	POS
43	23.3	28.7	0.8	NEG	44.1	27.9	1.35	POS	155	39.8	3.37	POS	109	2.79	Prolong	36.5	1.09	Normal	2.56	POS	107	NC	2.85	POS	36.4	C	1.13	NEG	2.52	POS	POS
44	47.6	42.8	1.1	NEG	57.4	35.3	1.39	POS	109	47	2.01	POS	40.2	1.03	Normal	37	1.11	Normal	0.93	NEG	37.7	C	1.00	NEG	33	C	1.03	NEG	0.97	NEG	NEG
45	76.1	52.3	1.3	POS	48.9	34.9	1.14	NEG	45.9	38.5	1.03	NEG	48.5	1.25	Normal	37	1.11	Normal	1.13	NEG	42.9	NC	1.14	POS	33.5	C	1.04	NEG	1.09	POS	NEG
46	141	59.4	2.1	POS	68.5	34.9	1.6	POS	84	39.6	1.84	POS	94.8	2.44	Prolong	47.9	1.43	Prolong	1.70	POS	80.6	NC	2.14	POS	37	NC	1.15	POS	1.86	POS	POS
47	40.7	39.4	1	NEG	31.6	28.1	0.96	NEG	38.6	29.7	1.13	NEG	35.4	0.91	Normal	29.8	0.89	Normal	1.02	NEG	35.7	C	0.95	NEG	30	C	0.93	NEG	1.02	NEG	NEG
48	32.1	36.9	0.9	NEG	36.9	29.9	1.06	NEG	43.7	33.4	1.13	NEG	36.3	0.93	Normal	29.1	0.87	Normal	1.07	NEG	36.2	C	0.96	NEG	29.9	C	0.93	NEG	1.03	NEG	NEG
49	29.4	37	0.8	NEG	29.4	28.5	0.88	NEG	28	31.5	0.77	NEG	33	0.85	Normal	31.5	0.94	Normal	0.90	NEG	34.4	C	0.91	NEG	30.9	C	0.96	NEG	0.95	NEG	NEG
50	37.2	36	1	NEG	31.1	27.1	0.98	NEG	39.1	29.7	1.14	NEG	35.7	0.92	Normal	31.6	0.95	Normal	0.97	NEG	36	C	0.96	NEG	31.2	C	0.97	NEG	0.98	NEG	NEG
51	25.6	24.7	1	NEG	34.2	28.7	1.02	NEG	42.7	31.9	1.16	NEG	28.8	0.74	Normal	22.7	0.68	Normal	1.09	NEG	30.7	C	0.82	NEG	25.4	C	0.79	NEG	1.03	NEG	NEG
52	31.6	51	0.6	NEG	31.6	30.7	0.88	NEG	33.5	31.5	0.92	NEG	36.2	0.93	Normal	37.2	1.11	Normal	0.84	NEG	37.7	C	1.00	NEG	33.8	C	1.05	NEG	0.95	NEG	NEG

percentile cutoff 99th

	SCT				dRVVT-HemosIL				dRVVT-Hemoclot				APTT-based																		
No.	screen (sec.)	confirm (sec.)	ratio	result	screen (sec.)	confirm (sec.)	ratio	result	screen (sec.)	confirm (sec.)	ratio	result	screen (sec.)	screen ratio	result	confirm (sec.)	confirm ratio	result	screen/ confirm ratio	result	Mixing screen (sec.)	NC/ C	Mixing ratio screen	result	Mixing confirm (sec.)	NC/ C	Mixing ratio confirm	result	Mixing screen/ confirm ratio	result	ISTH method
53	38.4	36.8	1.1	NEG	33.7	30.2	0.96	NEG	37.6	32.2	1.01	NEG	37	0.95	Normal	30.2	0.90	Normal	1.05	NEG	36.6	C	0.97	NEG	30.4	C	0.95	NEG	1.03	NEG	NEG
54	34.8	39.7	0.9	NEG	30.1	28.7	0.9	NEG	32.1	29	0.96	NEG	36.8	0.95	Normal	32.7	0.98	Normal	0.97	NEG	37.5	C	1.00	NEG	32.3	C	1.00	NEG	0.99	NEG	NEG
55	39.6	39.7	1	NEG	39.1	29.3	1.14	NEG	37	31	1.03	NEG	39.3	1.01	Normal	33	0.99	Normal	1.02	NEG	37.7	C	1.00	NEG	31.2	C	0.97	NEG	1.03	NEG	NEG
56	50	50.4	1	NEG	35	31.7	0.95	NEG	39.6	36.3	0.95	NEG	38.6	0.99	Normal	33.8	1.01	Normal	0.98	NEG	37.7	C	1.00	NEG	32.4	C	1.01	NEG	0.99	NEG	NEG
57	32.8	34.3	1	NEG	29.2	28.7	0.87	NEG	32.2	31.1	0.90	NEG	37.1	0.95	Normal	29.5	0.88	Normal	1.08	NEG	35.6	C	0.95	NEG	29.7	C	0.92	NEG	1.02	NEG	NEG
58	34.6	44	0.8	NEG	40.7	34.7	1	NEG	41	38.6	0.92	NEG	37.9	0.98	Normal	33.3	1.00	Normal	0.98	NEG	36.4	C	0.97	NEG	31.8	C	0.99	NEG	0.98	NEG	NEG
59	32.3	35.1	0.9	NEG	35.9	30	1.03	NEG	39.4	35.7	0.96	NEG	35.8	0.92	Normal	30.6	0.92	Normal	1.01	NEG	36.7	C	0.97	NEG	30.7	C	0.96	NEG	1.02	NEG	NEG
60	38.9	40.1	1	NEG	31.2	26.8	1	NEG	40.3	29.6	1.18	NEG	38.7	1.00	Normal	33	0.99	Normal	1.01	NEG	37.9	C	1.01	NEG	32.4	C	1.01	NEG	1.00	NEG	NEG
61	27.9	35.8	0.8	NEG	32.4	27.3	1.02	NEG	41	30.3	1.17	NEG	28.6	0.74	Normal	27.5	0.82	Normal	0.89	NEG	30.5	C	0.81	NEG	28.1	C	0.87	NEG	0.93	NEG	NEG
62	43.2	41.1	1.1	NEG	42.6	32.2	1.13	NEG	45.3	35.3	1.11	NEG	41.3	1.06	Normal	35.6	1.07	Normal	1.00	NEG	39.6	C	1.05	NEG	33.5	C	1.04	NEG	1.01	NEG	NEG
63	50.6	48.7	1.1	NEG	36.7	30.3	1.04	NEG	43	34.4	1.08	NEG	42.5	1.09	Normal	37	1.11	Normal	0.99	NEG	39.1	C	1.04	NEG	33.7	C	1.05	NEG	0.99	NEG	NEG
64	25.1	37.6	0.7	NEG	29.3	27.8	0.9	NEG	40.3	30.2	1.16	NEG	26.6	0.68	Normal	26.3	0.79	Normal	0.87	NEG	28.8	C	0.76	NEG	27.3	C	0.85	NEG	0.90	NEG	NEG
65	35.6	38.4	0.9	NEG	26	26.5	0.84	NEG	27.5	28.3	0.84	NEG	39.8	1.02	Normal	34.9	1.04	Normal	0.98	NEG	38.4	C	1.02	NEG	33.3	C	1.04	NEG	0.98	NEG	NEG
66	36.3	35.2	1	NEG	34.3	27.9	1.05	NEG	45.3	32.9	1.19	NEG	35.2	0.91	Normal	31.3	0.94	Normal	0.97	NEG	36.4	C	0.97	NEG	31.3	C	0.97	NEG	0.99	NEG	NEG
67	28.8	35.2	0.8	NEG	30.8	26.8	0.98	NEG	37.7	31.2	1.05	NEG	32.5	0.84	Normal	27.9	0.84	Normal	1.00	NEG	34.2	C	0.91	NEG	28.8	C	0.90	NEG	1.01	NEG	NEG
68	43.5	43.2	1	NEG	34.6	30.2	0.98	NEG	39.1	32.8	1.03	NEG	42.3	1.09	Normal	37.4	1.12	Normal	0.97	NEG	39	C	1.04	NEG	33.6	C	1.05	NEG	0.99	NEG	NEG
69	32.6	34	1	NEG	33	29.4	0.96	NEG	37.7	33.7	0.97	NEG	33.6	0.86	Normal	29.4	0.88	Normal	0.98	NEG	34.6	C	0.92	NEG	29.7	C	0.92	NEG	0.99	NEG	NEG
70	42.3	42.1	1	NEG	35.4	37.8	1.09	NEG	40.9	35.2	1.01	NEG	44.2	1.14	Normal	38.3	1.15	Normal	0.99	NEG	39.5	C	1.05	NEG	33.7	C	1.05	NEG	1.00	NEG	NEG
71	39.5	67.8	0.6	NEG	35.5	33.6	0.91	NEG	35.7	37.2	0.83	NEG	41.5	1.07	Normal	43.4	1.30	Prolong	0.82	NEG	37.8	C	1.00	NEG	35.2	C	1.10	NEG	0.92	NEG	NEG
72	38.1	38.7	1	NEG	31.8	28.3	0.96	NEG	41	33.8	1.05	NEG	44.7	1.15	Normal	34.4	1.03	Normal	1.12	NEG	38.7	C	1.03	NEG	32.8	C	1.02	NEG	1.01	NEG	NEG
73	51.9	47.5	1.1	NEG	46.2	35.3	1.12	NEG	59	42	1.22	NEG	44.1	1.13	Normal	38.5	1.15	Normal	0.98	NEG	40.4	C	1.07	NEG	34.4	C	1.07	NEG	1.00	NEG	NEG
74	38.8	38.2	1	NEG	40.3	30.9	1.12	NEG	46.1	37.2	1.07	NEG	36.7	0.94	Normal	33.7	1.01	Normal	0.94	NEG	37.2	C	0.99	NEG	32.5	C	1.01	NEG	0.98	NEG	NEG
75	41.3	39.8	1	NEG	40.8	31.2	1.12	NEG	38.7	32.2	1.04	NEG	38.4	0.99	Normal	33.1	0.99	Normal	1.00	NEG	37	C	0.98	NEG	31.6	C	0.98	NEG	1.00	NEG	NEG
76	30.1	32.8	0.9	NEG	30.4	25.4	0.99	NEG	34.4	28.2	1.06	NEG	33.1	0.85	Normal	30.1	0.90	Normal	0.95	NEG	34.7	C	0.92	NEG	30.1	C	0.94	NEG	0.98	NEG	NEG
77	44.4	44.5	1	NEG	42.7	31.6	1.16	NEG	44.8	34.8	1.12	NEG	40.8	1.05	Normal	34	1.02	Normal	1.03	NEG	38.5	C	1.02	NEG	32.6	C	1.01	NEG	1.01	NEG	NEG
78	45.9	42.4	1.1	NEG	34.5	30	0.99	NEG	35.7	31.3	0.99	NEG	41.1	1.06	Normal	34.2	1.02	Normal	1.03	NEG	37.7	C	1.00	NEG	32	C	1.00	NEG	1.01	NEG	NEG
79	31.9	33.7	1	NEG	34.9	28.6	1.05	NEG	41.3	32.5	1.10	NEG	35	0.90	Normal	30.1	0.90	Normal	1.00	NEG	35.8	C	0.95	NEG	30.4	C	0.95	NEG	1.01	NEG	NEG
80	31.8	34	0.9	NEG	27.9	26.5	0.9	NEG	32	29	0.96	NEG	32.7	0.84	Normal	28.5	0.85	Normal	0.99	NEG	31.5	C	0.84	NEG	28.6	C	0.89	NEG	0.94	NEG	NEG
81	34.5	51.7	0.7	NEG	38.7	33.2	1	NEG	42.1	42.1	0.87	NEG	39.4	1.01	Normal	39.2	1.17	Normal	0.86	NEG	37.6	C	1.00	NEG	35.1	C	1.09	NEG	0.91	NEG	NEG
82	32.6	34	1	NEG	33	29.4	0.96	NEG	34.2	31.4	0.94	NEG	32.5	0.84	Normal	28.2	0.84	Normal	0.99	NEG	33.5	C	0.89	NEG	28.9	C	0.90	NEG	0.99	NEG	NEG
83	30.9	37	0.8	NEG	31.1	28.1	0.95	NEG	36.2	29.1	1.08	NEG	30.7	0.79	Normal	27.1	0.81	Normal	0.97	NEG	30.9	C	0.82	NEG	26.9	C	0.84	NEG	0.98	NEG	NEG
84	46.9	39.3	1	NEG	40.7	29.9	1.17	NEG	49	32.9	1.29	POS	42	1.08	Normal	30.9	0.93	Normal	1.17	NEG	38.8	C	1.03	NEG	30.5	C	0.95	NEG	1.09	NEG	NEG
85	40.1	46.2	0.9	NEG	34.2	30.6	0.96	NEG	37.7	32.9	0.99	NEG	34.5	0.89	Normal	30.8	0.92	Normal	0.96	NEG	35.9	C	0.95	NEG	30.7	C	0.96	NEG	1.00	NEG	NEG
86	39.9	37	1.1	NEG	30	27	0.95	NEG	33.9	29.2	1.01	NEG	37.2	0.96	Normal	32.2	0.96	Normal	0.99	NEG	37.2	C	0.99	NEG	31.6	C	0.98	NEG	1.00	NEG	NEG
87	40.8	40.8	1	NEG	36.8	28.4	1.11	NEG	38.4	30.6	1.09	NEG	40.4	1.04	Normal	35	1.05	Normal	0.99	NEG	39.5	C	1.05	NEG	33.5	C	1.04	NEG	1.01	NEG	NEG
88	46.5	39	1.2	NEG	41.7	31.9	1.12	NEG	38.9	33.2	1.02	NEG	46.9	1.21	Normal	36.4	1.09	Normal	1.11	NEG	40.9	C	1.09	NEG	33	C	1.03	NEG	1.06	NEG	NEG
89	47.5	50.6	1	NEG	35	32.4	0.93	NEG	43.9	35.8	1.06	NEG	41.3	1.06	Normal	38.3	1.15	Normal	0.93	NEG	38.7	C	1.03	NEG	34.1	C	1.06	NEG	0.97	NEG	NEG
90	47	50.3	0.9	NEG	38.3	33.6	0.98	NEG	36	37.6	0.83	NEG	43.3	1.11	Normal	35.7	1.07	Normal	1.04	NEG	41.2	C	1.09	NEG	33.6	C	1.05	NEG	1.05	NEG	NEG
91	38.7	52.7	0.7	NEG	44.6	35.3	1.08	NEG	57.7	40.3	1.24	POS	39.8	1.02	Normal	35.2	1.05	Normal	0.97	NEG	39.5	C	1.05	NEG	33.8	C	1.05	NEG	1.00	NEG	NEG
92	33.3	36.2	0.9	NEG	39.2	31.9	1.05	NEG	60.6	43.5	1.21	NEG	32	0.82	Normal	32.7	0.98	Normal	0.84	NEG	34.5	C	0.92	NEG	30.7	C	0.96	NEG	0.96	NEG	NEG
93	45.1	47.4	1	NEG	43.6	35.6	1.05	NEG	46.9	40.6	1.00	NEG	37.6	0.97	Normal	32.3	0.97	Normal	1.00	NEG	36.9	C	0.98	NEG	30.8	C	0.96	NEG	1.02	NEG	NEG
94	36.6	37.3	1	NEG	38.5	28.7	1.15	NEG	48.6	34.8	1.21	NEG	38	0.98	Normal	33.4	1.00	Normal	0.98	NEG	37.7	C	1.00	NEG	31.8	C	0.99	NEG	1.01	NEG	NEG
95	41.5	39.5	1.1	NEG	34	27.7	1.05	NEG	43.1	28.9	1.29	POS	38.6	0.99	Normal	34.1	1.02	Normal	0.97	NEG	38.2	C	1.01	NEG	32.7	C	1.02	NEG	1.00	NEG	NEG
96	38	38	1	NEG	36.4	30	1.04	NEG	45.1	40.1	0.97	NEG	52.1	1.34	Prolong	35.4	1.06	Normal	1.26	POS	44.6	NC	1.18	POS	31.9	C	0.99	NEG	1.19	POS	POS
97	26.9	30.1	0.9	NEG	30.8	29.4	0.9	NEG	30	31.7	0.82	NEG	30.3	0.78	Normal	26.1	0.78	Normal	1.00	NEG	32.5	C	0.86	NEG	27.7	C	0.86	NEG	1.00	NEG	NEG
98	41.3	44.3	0.9	NEG	33	31.1	0.91	NEG	32.7	32.9	0.86	NEG	37.9	0.98	Normal	35.1	1.05	Normal	0.93	NEG	38	C	1.01	NEG	33.2	C	1.03	NEG	0.98	NEG	NEG
99	40.4	78	0.5	NEG	39	38.6	0.87	NEG	46.9	50.6	0.80	NEG	38.8	1.00	Normal	40.2	1.20	Normal	0.83	NEG	36.2	C	0.96	NEG	33.3	C	1.04	NEG	0.93	NEG	NEG
100	49.2	51.1	1	NEG	29.2	28.2	0.89	NEG	31.4	29.3	0.93	NEG	46.8	1.20	Normal	44.8	1.34	Prolong	0.90	NEG	40.7	C	1.08	NEG	36.5	C	1.14	NEG	0.95	NEG	NEG
101	37	37.5	1	NEG	33.8	29.9	0.97	NEG	37.6	31.5	1.03	NEG	36.3	0.93	Normal	28	0.84	Normal	1.11	NEG	35.7	C	0.95	NEG	28.4	C	0.88	NEG	1.07	NEG	NEG
102	35.4	34.7	1	NEG	30.9	27.8	0.95	NEG	28.9	27.9	0.90	NEG	36.6	0.94	Normal	32.1	0.96	Normal	0.98	NEG	37.3	C	0.99	NEG	31.5	C	0.98	NEG	1.01	NEG	NEG
103	41.7	44.3	1	NEG	34	30.1	0.97	NEG	44.5	34.3	1.12	NEG	38	0.98	Normal	32.8	0.98	Normal	1.00	NEG	37.5	C	1.00	NEG	32	C	1.00	NEG	1.00	NEG	NEG
104	38.6	44.2	0.9	NEG	33.2	31.7	0.9	NEG	37.4	34.1	0.95	NEG	39.1	1.01	Normal	35	1.05	Normal	0.96	NEG	38.6	C	1.02	NEG	33	C	1.03	NEG	1.00	NEG	NEG

percentile cutoff 99th

	SCT				dRVVT-HemosIL				dRVVT-Hemoclot				APTT-based																			
No.	screen (sec.)	confirm (sec.)	ratio	result	screen (sec.)	confirm (sec.)	ratio	result	screen (sec.)	confirm (sec.)	ratio	result	screen (sec.)	screen ratio	result	confirm (sec.)	confirm ratio	result	screen/ confirm ratio	result	Mixing screen (sec.)	NC/ C	Mixing ratio screen	result	Mixing confirm (sec.)	NC/ C	Mixing ratio confirm	result	Mixing screen/ confirm ratio	result	ISTH method
105	38.7	40.5	1	NEG	32	30.9	0.89	NEG	36.4	34.3	0.92	NEG	36.5	0.94	Normal	33.1	0.99	Normal	0.95	NEG	37	C	0.98	NEG	32.8	C	1.02	NEG	0.96	NEG	NEG
106	38.7	36	1.1	NEG	36.2	28.6	1.08	NEG	41	32.1	1.11	NEG	39.9	1.03	Normal	31.4	0.94	Normal	1.09	NEG	38.5	C	1.02	NEG	30.9	C	0.96	NEG	1.06	NEG	NEG
107	49.2	51.1	1	NEG	29.2	28.2	0.89	NEG	32.8	29.9	0.95	NEG	47	1.21	Normal	43.8	1.31	Prolong	0.92	NEG	40.6	C	1.08	NEG	36.5	C	1.14	NEG	0.95	NEG	NEG
108	41.1	46.9	0.9	NEG	35.5	30.8	0.99	NEG	37.4	35.3	0.92	NEG	40.3	1.04	Normal	35.6	1.07	Normal	0.97	NEG	39.2	C	1.04	NEG	33.5	C	1.04	NEG	1.00	NEG	NEG
109	51.8	49.7	1.1	NEG	46.3	33.6	1.18	NEG	79.8	47.9	1.44	POS	54.6	1.41	Prolong	43.4	1.30	Prolong	1.08	NEG	44.9	NC	1.19	POS	36.3	C	1.13	NEG	1.06	NEG	NEG
110	38.5	38.8	1	NEG	40.7	32	1.09	NEG	42.8	36.8	1.01	NEG	36.2	0.93	Normal	31.7	0.95	Normal	0.98	NEG	35.9	C	0.95	NEG	30.3	C	0.94	NEG	1.01	NEG	NEG
111	93.6	39.7	2.4	POS	126	35.6	3.02	POS	240	56	3.71	POS	124	3.20	Prolong	50.5	1.51	Prolong	2.11	POS	95.7	NC	2.54	POS	40.2	NC	1.25	POS	2.03	POS	POS
112	42.9	54.4	0.8	NEG	64.6	66	0.84	NEG	74.6	115.7	0.56	NEG	57.1	1.47	Prolong	55.9	1.67	Prolong	0.88	NEG	32.8	C	0.87	NEG	32.5	C	1.01	NEG	0.86	NEG	NEG
113	54.8	39.6	1.4	POS	48.1	32.4	1.27	POS	53.8	36.8	1.27	POS	45.7	1.18	Normal	29.5	0.88	Normal	1.33	POS	40.2	C	1.07	NEG	29.7	C	0.92	NEG	1.16	POS	NEG
114	51	46.4	1.1	NEG	41.6	30.8	1.16	NEG	38	34.2	0.96	NEG	43.3	1.11	Normal	34.1	1.02	Normal	1.09	NEG	39.1	C	1.04	NEG	32.4	C	1.01	NEG	1.03	NEG	NEG
115	42.5	40.9	1.1	NEG	36.4	26.3	1.19	POS	35.3	29	1.06	NEG	38.4	0.99	Normal	33.7	1.01	Normal	0.98	NEG	37.6	C	1.00	NEG	31.7	C	0.99	NEG	1.01	NEG	NEG
116	56.5	42.7	1.3	POS	47.2	33.6	1.2	POS	39.2	35.9	0.95	NEG	50.6	1.30	Prolong	36.2	1.08	Normal	1.20	NEG	43.7	NC	1.16	POS	33.8	C	1.05	NEG	1.10	POS	NEG
117	94.1	48.3	2	POS	50.6	30.5	1.42	POS	68.7	41.6	1.43	POS	76.7	1.97	Prolong	51.6	1.54	Prolong	1.28	POS	65	NC	1.73	POS	38.2	NC	1.19	POS	1.45	POS	POS
118	141	44.1	3.2	POS	75.9	30.7	2.12	POS	78.2	32.6	2.08	POS	98.8	2.54	Prolong	32.7	0.98	Normal	2.60	POS	75.5	NC	2.00	POS	31.6	C	0.98	NEG	2.04	POS	POS
119	237	47.3	5	POS	115	33	2.98	POS	134	42.1	2.75	POS	120	3.09	Prolong	44.1	1.32	Prolong	2.34	POS	120	NC	3.18	POS	43	NC	1.34	POS	2.38	POS	POS
120	131	40.7	3.2	POS	60	28.4	1.81	POS	106	33.3	2.75	POS	95.6	2.46	Prolong	42.6	1.28	Normal	1.93	POS	77.8	NC	2.07	POS	35.7	C	1.11	NEG	1.86	POS	POS
121	39.1	39.8	1	NEG	29.6	29.5	0.86	NEG	38.4	33.2	1.00	NEG	35.3	0.91	Normal	30.6	0.92	Normal	0.99	NEG	36.8	C	0.98	NEG	30.9	C	0.96	NEG	1.02	NEG	NEG
122	48.6	41.2	1.2	NEG	34.8	31.1	0.96	NEG	41.7	35.1	1.03	NEG	42	1.08	Normal	33.5	1.00	Normal	1.08	NEG	39.1	C	1.04	NEG	32.3	C	1.00	NEG	1.03	NEG	NEG
123	47.4	47.9	1	NEG	37	29.9	1.06	NEG	32.8	31	0.92	NEG	40	1.03	Normal	33.2	0.99	Normal	1.04	NEG	39	C	1.04	NEG	32.2	C	1.00	NEG	1.03	NEG	NEG
124	37.9	37.8	1	NEG	27.6	26.7	0.89	NEG	30.1	28.4	0.92	NEG	37.1	0.95	Normal	33.2	0.99	Normal	0.96	NEG	37.1	C	0.99	NEG	31.7	C	0.99	NEG	1.00	NEG	NEG
125	36.1	39.2	0.9	NEG	32.5	28.2	0.99	NEG	32.8	29.9	0.95	NEG	41.9	1.08	Normal	35	1.05	Normal	1.03	NEG	39.6	C	1.05	NEG	33	C	1.03	NEG	1.02	NEG	NEG
126	48.9	56	0.9	NEG	35	31.8	0.94	NEG	36.3	35.6	0.88	NEG	44	1.13	Normal	39.7	1.19	Normal	0.95	NEG	39.9	C	1.06	NEG	34.8	C	1.08	NEG	0.98	NEG	NEG
127	35.2	37.5	1	NEG	31.5	45.4	0.59	NEG	39.3	30.6	1.11	NEG	33.8	0.87	Normal	28	0.84	Normal	1.04	NEG	34.9	C	0.93	NEG	28.6	C	0.89	NEG	1.04	NEG	NEG
128	60.1	65.7	0.9	NEG	44.9	33.3	1.16	NEG	41.4	38.2	0.94	NEG	61.2	1.57	Prolong	46	1.38	Prolong	1.14	NEG	47.8	NC	1.27	POS	37.6	NC	1.17	POS	1.08	NEG	NEG
129	52.3	44.1	1.2	NEG	47.2	30.3	1.33	POS	36.5	29.2	1.08	NEG	43.8	1.13	Normal	32.2	0.96	Normal	1.17	NEG	41.1	C	1.09	NEG	31.7	C	0.99	NEG	1.11	POS	NEG
130	75	36.1	2.1	POS	57.1	31	1.58	POS	77.7	34.1	1.98	POS	94.5	2.43	Prolong	37	1.11	Normal	2.20	POS	98.4	NC	2.61	POS	36.1	C	1.12	NEG	2.33	POS	POS
131	42.3	48	0.9	NEG	41.2	32.4	1.09	NEG	44.9	39.2	0.99	NEG	41.7	1.07	Normal	38.9	1.16	Normal	0.92	NEG	40.1	C	1.06	NEG	34.6	C	1.08	NEG	0.99	NEG	NEG
132	45.4	47.2	1	NEG	35.4	28.9	1.05	NEG	40.7	34.6	1.02	NEG	42.9	1.10	Normal	36	1.08	Normal	1.02	NEG	39.3	C	1.04	NEG	33.4	C	1.04	NEG	1.00	NEG	NEG
133	30.4	29.2	1.1	NEG	31.3	25.7	1.04	NEG	31	27	1.00	NEG	30.6	0.79	Normal	24.7	0.74	Normal	1.06	NEG	31.8	C	0.84	NEG	26.2	C	0.82	NEG	1.04	NEG	NEG
134	49	58.6	0.8	NEG	36.6	34.3	0.91	NEG	38.9	37.1	0.91	NEG	40.8	1.05	Normal	37.9	1.13	Normal	0.93	NEG	39.2	C	1.04	NEG	34.3	C	1.07	NEG	0.98	NEG	NEG
135	41.3	35.3	1.2	NEG	39	29.3	1.14	NEG	24.9	24.6	0.88	NEG	32.8	0.84	Normal	28.7	0.86	Normal	0.98	NEG	34.5	C	0.92	NEG	29.5	C	0.92	NEG	1.00	NEG	NEG
